# Predictive and prognostic factors influencing outcomes of rituximab therapy in systemic lupus erythematosus (SLE): A systematic review^☆^

**DOI:** 10.1016/j.semarthrit.2017.04.010

**Published:** 2017-12

**Authors:** Carmelo Pirone, Claudia Mendoza-Pinto, Daniëlle A. van der Windt, Ben Parker, Miriam O׳Sullivan, Ian N. Bruce

**Affiliations:** a Department of Internal Medicine and Medical Specialties, Rheumatology Unit, Sapienza University of Rome, Rome, Italy; b Systemic Autoimmune Disease Research Unit, Regional General Hospital 36-CIBIOR, Mexican Institute for Social Security, Puebla, México; c Arthritis Research UK Primary Care Centre, Research Institute for Primary Care & Health Sciences, Keele University, Keele, Staffordshire, UK; d NIHR Manchester Musculoskeletal Biomedical Research Unit, Central Manchester University Hospitals NHS Foundation Trust, Manchester Academic Health Science Centre, Manchester, UK; e Centre for Musculoskeletal Research, Faculty of Biology, Medicine and Health, The University of Manchester, Manchester Academic Health Science Centre, Manchester, UK; f Arthritis Research UK Centre for Epidemiology, Faculty of Biology, Medicine and Health, The University of Manchester, Manchester Academic Health Science Centre, Oxford Road, Manchester, UK

**Keywords:** Systemic lupus erythematosus, Rituximab, Systematic review, Prognosis

## Abstract

**Background:**

The clinical outcomes following rituximab (RTX) treatment in patients with systemic lupus erythematosus (SLE) is highly variable. We aimed to identify predictive and prognostic factors associated with RTX therapy outcomes in patients with SLE.

**Methods:**

Studies in adults and paediatric patients with SLE were included. We included randomized clinical trials (RCTs) for predictors of differential treatment effect and cohort studies for potential prognostic factors in patients treated with RTX (global clinical, cutaneous and renal either response or relapse, and side effects). Methodological quality was assessed using Cochrane Collaboration Risk of Bias tool and the Quality In Prognosis Studies Tool (QUIPS) for RCTs and cohort studies, respectively. The quality of subgroup analyses testing predictors of differential treatment response was also evaluated. A best evidence synthesis was performed using the Grading of Recommendations Assessment, Development, and Evaluation (GRADE) framework.

**Results:**

Sixteen articles were included (3 from 2 RCTs and 13 from 6 cohort studies). The overall quality of evidence (QoE) was low to very low (GRADE framework). QoE for predictive factors based on RCTs analysing sociodemographic variables, was rated very low due to the lack of interaction tests, limited power of subgroup analyses, study limitations, and imprecisions. Disease-related factors including clinical phenotype and severity, baseline anti-ENA antibodies and anti-Ro antibodies, interleukin (IL) 2/21 single nucleotide polymorphism (SNP), as well as post-RTX complete B-cell depletion and earlier B-cell repopulation showed some evidence for prognostic value, but were rated low to very low QoE because of early phase of investigation (exploratory analysis), insufficient adjustment for confounding in most studies, high risk of bias, inconsistency, and imprecisions.

**Conclusions:**

To date, studies addressing prognostic factors are hypothesis generating and cannot be used to make any specific recommendations for routine clinical practice. A number of potential predictors/prognostic factors were identified, which require to be validated as being specific for response to RTX therapy and to enable more personalised use of this agent.

## Introduction

Personalised medicine research is emerging across a number of medical disciplines [1] and if successful will enable us to move from “all comer” or “empirical” medicine to a more targeted approach thus making the best decisions for individuals or groups of similar patients [2], [3]. A stratified medicine approach requires testing of patients for the presence of factors considered predictive of an improved treatment response (more benefit, less harm, or both) compared to other (active) treatment options. Thus, the ability to target optimal therapy to the right patient will have an impact on healthcare delivery, quality, and costs of care.

Systemic lupus erythematosus (SLE) is an autoimmune disease characterised by loss of tolerance to nucleic acids and highly diverse clinical manifestations [4]. B-cell depletion with the anti-CD20 monoclonal rituximab (RTX) has been found to be effective in a number of autoimmune conditions including rheumatoid arthritis (RA) [5]. Successful use of RTX in patients with SLE has been reported in a number of open-label cohorts studies [6], and a recent meta-analysis supported its effectiveness in refractory SLE [7]. Two RCTs of RTX in patients with SLE (EXPLORER [8] and LUNAR [9]) did not, however, achieve their primary end points. Some researchers have cogently argued that various aspects of trial design could account for these apparent failures [6]. RTX is therefore now an established drug used in the treatment of SLE and its use is supported in several guidelines [10], [11]. However, variability in biological and/or clinical response to RTX has been reported in a number of studies [12], [13]. In addition to study design issues, heterogeneity of the SLE population is also likely to contribute to these variable results, suggesting a single therapy or therapeutic approach may not be equally effective in all patients with SLE. A better understanding of why some patients respond better than others to RTX and in particular which factors are associated with better responses (or more adverse events) is therefore important to optimise and better target the use of this therapy to improve patient outcomes.

The objectives of this systematic review therefore were (1) to identify predictors of differential response (moderators) to RTX therapy for SLE in RCTs and (2) to identify prognostic factors associated with outcomes following RTX therapy in cohort studies of patients with SLE.

## Methods

### Literature search

Studies were identified through a systematic literature search in the following databases: MEDLINE via Ovid (1946 to December 2015), EMBASE via Ovid (1974 to December 2015), The Cochrane Central Register of Randomized Controlled Trials (CENTRAL-The Cochrane Library) via Ovid (to December 2015), and Web of Science (to December 2015). Additional studies were identified through a review of the included studies׳ reference lists. To ensure proper interpretation of the results by our team, publication language was restricted to English, Italian, or Spanish. The search strategies used for Ovid MEDLINE^®^ and applied to other databases in the literature are available in Supplementary File A, Table A.1.

### Selection criteria

Publications were included in the review if they met the following inclusion criteria: (1) RCTs and quasi-randomized studies in all different phases that compared RTX therapy vs control in SLE patients and (2) prospective or retrospective cohort studies, which have included at the beginning of follow-up not less than 30 patients. We decide to include observational cohorts with a minimum of 30 patients in our systematic review because, for prognostic factors, there are accepted methods for calculating the sample size for binary or continuous outcomes [14], and if confounding factors also are considered, smaller sample size would have been irrelevant for our purposes. We also excluded review articles, opinion papers, letters to the editor, case reports, case series, or conference abstracts. RCTs or cohort studies reporting outcomes for RTX therapy as a combination therapy with immunosuppressant agents (except when RTX was added to previous stable dose treatment) were also excluded.

### Study screening

References and abstracts identified by the search were imported into Reference Manager (RefMan) Version 12 and duplicates were removed. The resulting titles and abstracts were, independently, reviewed by C.P. and C.M.P. If titles and abstract did not give enough information to judge eligibility, full manuscripts were procured and independently reviewed by two reviewers (C.P. and C.M.P.). The full text of each article was then tested against all inclusion and exclusion criteria. The review team made every effort to identify multiple publications from a single study to obtain all relevant information from trials and cohorts. Disagreements regarding eligibility were resolved through discussion or by a third reviewer (I.N.B. or B.P.) if necessary. The bibliographies of all included studies were manually screened for additional articles of interest.

### Data extraction

Standardised data extraction forms were used to extract the following study details for RCTs: author identification, year of publication, setting, number of patients included, intervention, and control treatment including dose and administration details, duration of follow-up, differential treatment predictors, or subgroups analysis and relevant outcomes; and for prognostic cohort studies: study design, setting, study duration, number of patients included, prognostic factors, relevant outcomes, and adjustment for confounders. Definitions for prognostic factors or outcomes were taken from the included publications (Supplementary File A, Table A.2). Data extraction was done independently by two reviewers (C.P. and C.M.P.). When available, estimates of treatment effects for patient subgroups in RCTs, and associations of prognosis factors with treatment outcome in cohorts were extracted from each published report. Where insufficient information on these estimates was provided in original reports, where possible we used available data to calculate relative risks and corresponding 95% confidence interval (95% CI) using methods recommended in the Cochrane Handbook for Systematic Reviews of interventions [15]. Moderators or prognostic factors had to have been measured either at baseline or during RTX therapy.

### Methodological quality assessment

In accordance with PRISMA guidelines we assessed methodological quality of included studies [16]. The methods used in this for quality appraisal are described in detail elsewhere in this issue [17], but in short, the Cochrane risk of bias tool [18], [19], [20] was used for RCTs, while the Quality In Prognosis Studies Tool was used to assess risk of bias in cohort studies (Supplementary File A, Table A.3). The quality of subgroup (moderation) analyses in RCTs was also evaluated using criteria proposed by Pincus et al. [19]. Two reviewers (C.M.P. and M.O.S.) independently rated the methodological quality of the selected studies. The two reviewers discussed disagreement about whether a criterion was met, which was resolved by consensus.

### Data synthesis

Due to the expected heterogeneity of selected studies, we performed a narrative best evidence synthesis to summarise evidence for potential predictors from RCTs and prognostic factors from cohort studies, which takes into account the strength of the association and the methodological quality of the studies. We identified three PICO (Population, Intervention, Comparator, Outcome) questions [21] regarding potential predictors of the effect of RTX and four PICO questions regarding prognostic factors to structure the evidence synthesis. The overall quality of evidence (QoE) was assessed for each PICO question for RCTs using GRADE (Grading of Recommendations, Assessment, Development, and Evaluation) for RCTs [22] and the GRADE adaptation for prognostic evidence [23] (Supplementary File A, Table A.4). The PICO comparison (C) category was not applicable and dropped for cohort studies.

We used Review Manager (RevMan) to summarise the data and GRADE profiler (GRADEpro) software to produce the GRADE profile [24]. More details about GRADE evaluation can be found elsewhere (Supplementary File A, Tables A.5 and A.6) [17].

## Results

### Literature search

**Fig. 1:**
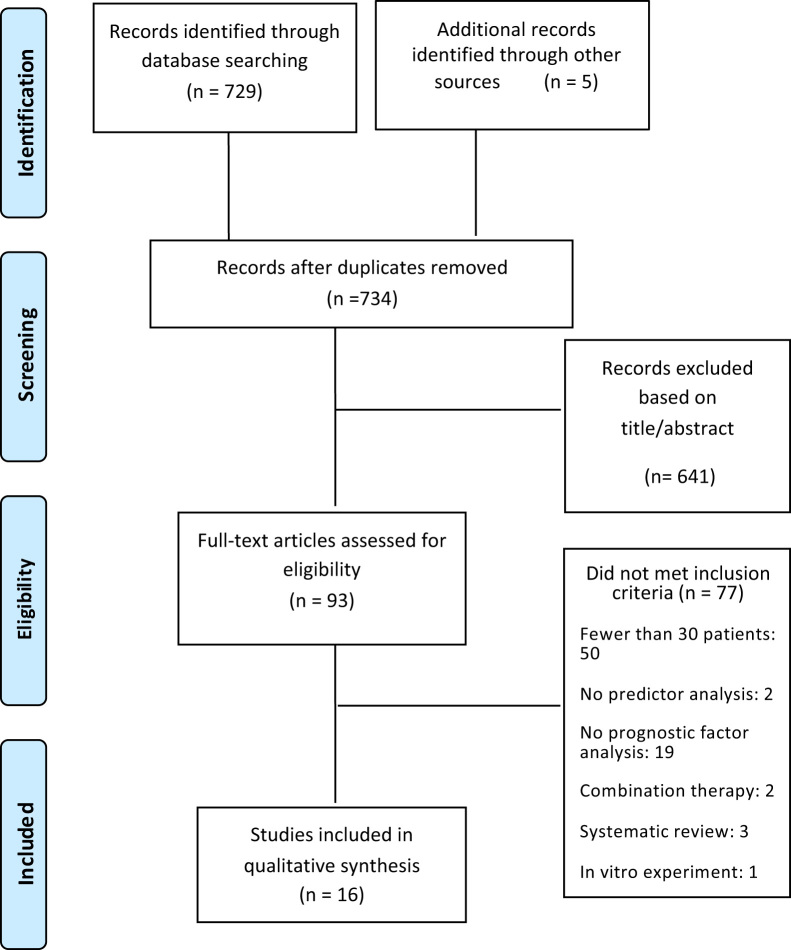
Study flow diagram detailing the literature search.

The electronic searches resulted in 734 records after exclusion of duplicates, and 94 full articles were assessed for eligibility (see PRISMA flow chart, Fig. 1). A total of 16 articles met the eligibility criteria and were included in the review (3 papers from 2 RCTs and 13 papers from 6 cohort studies). A list of excluded studies and the reason for exclusion are available in Supplementary File A, Table A.7.

### Study characteristics

The characteristics of the included studies are given in Table 1, Table 2 (RCTs and cohort studies, respectively). We included analyses from two RCTs [8], [9], one of which was reported in a separate secondary paper with a biomarker end point [25]. One RCT reported on multiple potential predictors of the effect of RTX [8]. From two RCTs, we extracted data only in the RTX arm when specific possible predictors for this therapy were only examined in this group; we displayed these results in the tables for prognostic factors used in cohort studies [9], [25].

**Table 1:** Characteristics of RCTs evaluating the effectiveness of rituximab in patients with SLE

Study ID	PICO	Setting	Population	No. of patients	Intervention	Follow-up	Possible predictor	Outcomes
Merrill et al. [8]^a^	1	International	Moderately to severely active SLE	257	Rituximab 1000 mg (*n* = 169) or placebo (*n* = 88) on days 1, 15, 168, and 182	52 wk	Age	Major clinical response
	2						Gender	Partial clinical response
	3						Race	Overall response
							Assigned prednisone dose	No response
							Background immunosuppressant	
							Duration of lupus	
							Baseline BILAG A score	
							Baseline BILAG-defined mucocutaneous or musculoskeletal system involvement	
Rovin et al. [9]	3	International	LN III, IV	144	Rituximab 1 g on days 1, 15, 168, and 182 + MMF 3 g/d (*n* = 72) or MMF (3 g/d) (*n* = 72)	52 wk	B cells at baseline	Overall response
						78 wk	B-cell depletion	
Tew et al. [25]^a^	3	International	Moderately to severely active SLE	257	Rituximab 1000 mg (*n* = 169) or placebo (*n* = 88) on days 1, 15, 168, and 182	52 wk	Autoantibodies	Normalisation of complement and anti-dsDNA
							B-cell levels	
							Interferon signature	

Studies are listed in alphabetical order. HACA, human antichimeric antibody; IVC, intravenous cyclophosphamide; LN, lupus nephritis; MMF, mycophenolate mofetil; PICO, Population, Intervention, Comparator, Outcome (number of PICO question); RCT, randomized clinical trial.

a Multiple articles on partially the same trial cohort.

**Table 2:** Characteristics of studies on prognostic factors

Study ID	Setting	PICO	Design	No. of patients	Dose of rituximab	Follow-up	Possible predictor	Outcomes	Adjustment for confounders
Carter et al. [26]^a^	UK	7	Retrospective cohort	35	1 g × 2 plus 750 mg IVC	66 wk^e^	Serum BAFF levels	Clinical relapse	Not indicated
								Changes in B cells	
								High anti-dsDNA	
Catapano et al. [29]	UK	7	Retrospective cohort	31	375 mg/m^2^/wk × 4 or 1000 mg × 2	30 mo^e^	Serologic features	Response	Not indicated
								Relapse	
Dias et al. [30]^a^	UK	7	Retrospective cohort	98	1 g × 2 plus 750 mg IVC	5 y^f^	Longer duration of BCD	Clinical improvement	Sociodemographic, clinical, and serological features
Fernandez-Nebro et al. [31]	Spain	6	Retrospective cohort	116	375 mg/m^2^/wk × 4 or 1000 mg × 2	20 mo^f^	Disease-related variables	Clinical response	Sociodemographic, clinical, and treatment variables
								Adverse events	
Jónsdóttir et al. [32]a,b	International	6	Retrospective cohort	43	375 mg/m^2^/wk × 4 or 1000 mg × 2 plus 500–1000 mg IVC	6 mo	LN histopathological class	Renal improvement	Not indicated
Lazarus et al. [27]^a^	UK	7	Retrospective cohort	61	1 g of rituximab × 2 plus 750 mg IVC	52 wk	Anti-dsDNA antibody levels at baseline	Clinical relapse	Not indicated
							B-cell repopulation		
Lindholm et al. [28]	Sweden	6	Retrospective cohort	33	375 mg/m²/wk × 4 wk	22 mo^e^	LN duration	Renal response	Not indicated
		7					Baseline serum creatinine		
							Baseline proteinuria		
							Baseline anti-dsDNA		
							Baseline detectable B cell		
Marquez et al. [36]^c^	Spain	5	Prospective cohort	84	375 mg/m^2^/wk × 4 or 1000 mg × 2	6 mo	Genetic factors	Clinical complete response	Sociodemographic and concomitant therapies
Ng et al. [33]^a^	UK	7	Retrospective cohort	32	1 g of rituximab × 2 plus 750 mg IVC	39 mo^e^	Anti-ENA	Clinical flare	Performed but unknown confounders
Robledo et al. [34]^c^	Spain	5	Prospective cohort	81	375 mg/m^2^/wk × 4 or 1000 mg × 2	6 mo	Genetic factors	Clinical response	Not indicated
Robledo et al [35]^c^	Spain	5	Prospective cohort	83	375 mg/m^2^/wk × 4 or 1000 mg × 2	6 mo	Genetic factors	Clinical response	Not indicated
Vital et al. [13]^d^	UK	7	Prospective cohort	39	1000 mg × 2	6 mo	Anti-ENA	Clinical response	Not indicated
							B-cell depletion	Clinical relapse	
							B-cell repopulation		
Vital et al. [37]^d^	UK	6	Retrospective cohort	82	1000 mg × 2	6 mo	Cutaneous phenotype	Mucocutaneous response	Not indicated
		7					Autoantibodies	Cutaneous flare	
							Complement		
							B-cell depletion		

Studies are listed in alphabetical order. Anti-ENA, anti-extractable nuclear antigen; BAFF, B-cell-activating factor; HCQ, hydroxychloroquine; IVC, intravenous cyclophosphamide; LN, lupus nephritis; MLN, membranous lupus nephritis; PICO, Population, Intervention, Comparator, Outcome (number of PICO question); RCT, randomized clinical trial; SELENA-SLEDAI: Safety of Estrogens in Lupus Erythematosus National Assessment-SLE disease activity index; UK, United Kingdom.

a,b,c,d Multiple articles on partially the same cohort.

e Median.

f Mean.

Across RCTs and cohorts, five studies included participants aged 15–17 years [8], [9], [26], [27], [28], while seven studies only included patients aged 18 years or older [29], [30], [31], [32], [33]. In five studies, mean age was not reported [13], [34], [35], [36], [37]. The follow-up duration varied from 24 to 78 weeks (6–18 months) for RCTs and 6–60 months for cohort studies. One RCT (or subgroup analyses) included only patients with active LN [9] and one evaluated patients with extra-renal manifestations [8]. Two cohort studies took into account active LN [9], [28], [32], one study evaluated mucocutaneous either response or flare [37] and the remaining studies analysed global clinical response or relapse. No cohort studies were identified that described the association of sociodemographic factors with outcomes in SLE patients with RTX (PICO 4).

### Characteristics of possible predictive or prognostic factors and outcomes to RTX

Predictive or prognostic factors were grouped into four categories—sociodemographic, genetic, disease-related, and laboratory biomarkers. Outcomes were evaluated as follows: global, renal, or cutaneous response/remission were evaluated by 12 studies [8], [9], [13], [27], [28], [30], [31], [32], [34], [35], [36], [37]; global, renal, or cutaneous relapse/ flare were reported in five studies [26], [27], [29], [33], [37]; harms including adverse events were evaluated by two studies [13], [31]; and changes in biomarkers were reported in four studies [9], [13], [25], [26].

### Methodological quality of included studies

#### Risk of bias in RCTs

**Fig. 2:**
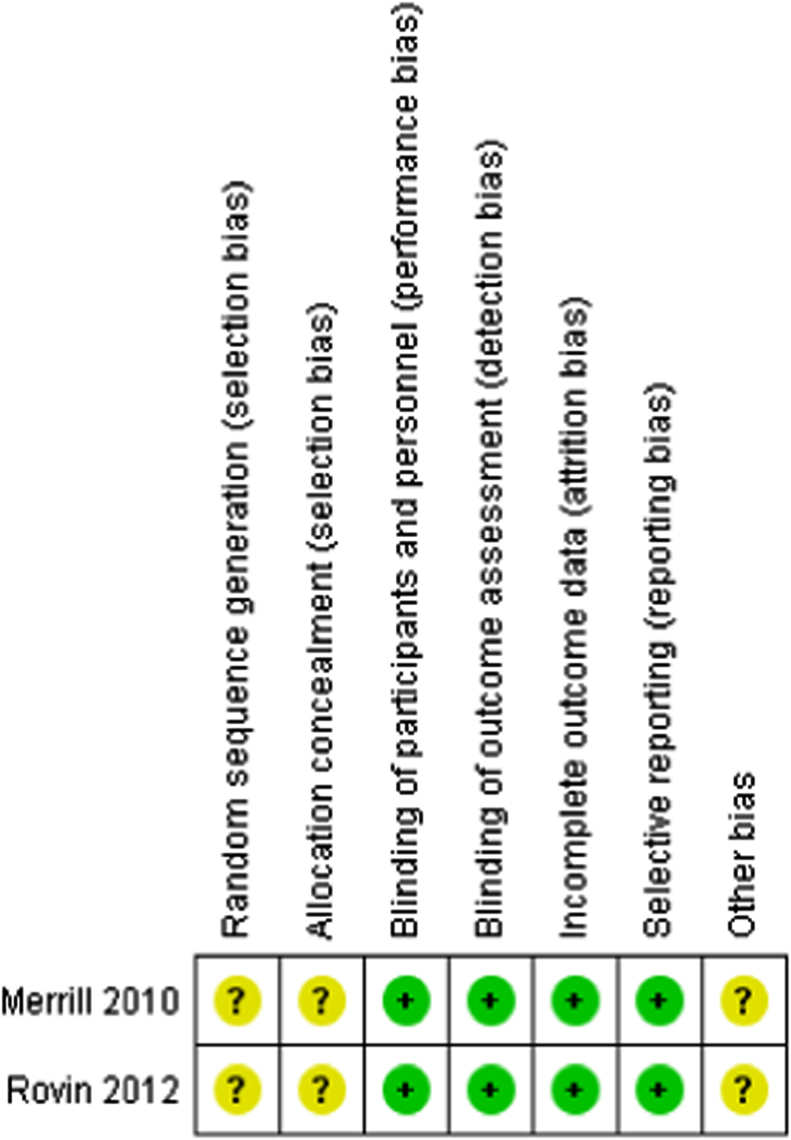
Risk of bias summary of RCTs included.

Risk of bias assessment was based on the main results paper of the included RCTs. The methods of randomization and allocation concealment were unclear (high risk) in both RCTs. Two trials were described as double blinded (participant and outcome assessment) and rated as low risk of performance and detection bias [8], [9]. These trials included an intention-to-treat (ITT) analysis, had no evidence of selective outcome reporting, and dropout rate analyses were adequately presented (low risk of attrition bias). The RCTs either declared sponsorship by a pharmaceutical industry company, or included an author who declared pharmaceutical company affiliation; these were judged as carrying high-risk bias related to the funding source (Fig. 2).

#### Quality of subgroups (moderation) analysis

At least nine subgroup analyses were conducted in one RCT [8], without providing a clear rationale a priori for most of them. None of the subgroup analyses provided confirmatory evidence with one analysis providing exploratory evidence only (Supplementary File B, Table B.1).

#### Risk of bias in prognostic factor studies based on observational cohorts

The overall methodological quality of six studies scored “moderate”, seven studies scored “low”, and no study was judged as “high” quality (Supplementary File B, Table B.2). Due to lack of reporting on key characteristics of the source population (“study participation”) and of participants loss to follow-up (“study attrition”), bias could not be ruled out, therefore studies were classified as “moderate” (*n* = 4) or “high” risk (*n* = 9) of selection bias. In 10 studies, measurement of prognostic factors and outcomes were performed in a similar, valid, and reliable way for all participants (“low” to “moderate” risk of bias), although, in three studies outcomes measurements were based on clinical judgement instead of valid criteria [34], [35], [36], resulting in high risk of bias for these domains. The statistical analysis, model-building process, or completeness of reporting was judged to be inadequate in all studies (“moderate” to “high” risk of bias). In particular, there was insufficient description of how factors were selected for inclusion in the multivariable analysis (Supplementary File D, Table D.1–D.7).

### Synthesis of evidence

#### Sociodemographic factors (PICO 1 and 4)

##### Age, gender, and race

One RCT [8] found no association between age or gender and response to RTX. In a pre-specified secondary analysis being of African American/Hispanic race/ethnicity showed a larger effect of RTX on no clinical response at week 52 compared to those of other ethnic origin. No differences were seen for secondary end points such as quality of life or major clinical response with a prednisone dosage of <10 mg/d in the same subgroup (low QoE, Supplementary File C).

#### Genetic factors (PICO 5)

In one cohort study [36], both IL2/IL21 (rs6822844) GG genotype and G allele were significantly associated with clinical response when adjusted for age, gender, and concomitant therapies (OR = 6.43, 95% CI: 1.42–21.07) (Table 3). The QoE for this association was downgraded to low due to the early phase of investigation designed to generate hypotheses and imprecision of the results but also upgraded given the strength of the association (Supplementary File D, Table D.1).

**Table 3:** Summary of evidence for factors associated with global clinical response

Factor identified		Study	*N*	Outcome	Unadjusted ES (95% CI)/univariable analysis	*p* Value	Adjusted ES (95% CI)	*p* Value	Overall quality
Genetic	-174 IL-6 (rs1800795) SNP GG vs GC genotype	Robledo et al. [34]	40/36	Complete or partial response	36 (90.0%) vs 32 (88.9%)	0.87	NP	NP	+
					RR = 1.01 (0.87–1.18)				
	-174 IL-6 (rs1800795) SNP GG vs CC genotype	Robledo et al. [34]	40/8	Complete or partial response	36 (90.0%) vs 5 (62.5%)	0.34	NP	NP	+
					RR = 1.26 (0.77–2.03)				
	-174 IL-6 (rs1800795) SNP GC vs CC genotype	Robledo et al. [34]	36/8	Complete or partial response	32 (88.9%) vs 5 (62.5%)	0.21	NP	NP	+
					RR = 1.42 (0.82–2.46)				
	FCGR3A-158 SNP VV vs FF genotype	Robledo et al. [35]	13/44	Complete or partial response	12 (92.3%) vs 36 (81.8%)	0.25	NP	NP	+
					RR = 1.13 (0.91–1.39)				
	FCGR3A-158 SNP FV vs VV genotype	Robledo et al. [35]	24/13	Complete or partial response	23 (95.8%) vs 12 (92.3%)	0.68	NP	NP	+
					RR = 1.04 (0.87–1.24)				
	FCGR3A-158 SNP FV vs FF genotype	Robledo et al. [35]	24/44	Complete or partial response	23 (95.8%) vs 36 (81.8%)	0.056	NP	NP	+
					RR = 1.17 (0.99–1.37)				
	IL2/IL21 SNP (rs6822844) GG vs GT genotype	Marquez et al. [36]	66/18	Complete or partial response	61 (92.4%) vs 12 (66.7%); RR = 1.39 (0.99–1.94)	0.055	**OR = 6.43 (1.42–21.07)**	**0.016**	++

Disease related	Baseline SLEDAI	Fernandez-Nebro et al. [31]	116	Complete/partial remission	OR = 1.1 (1.03–1.2)	0.001	**OR= 1.1, (1.04–1.16)**	**0.001**	+
	Previous discoid rash	Fernandez-Nebro et al. [31]	116	Complete or partial remission	**OR = 4.4 (1.2–15.8)**	**0.025**	NS	0.08	+
	Previous severe haematologic disorder	Fernandez-Nebro et al. [31]	116	Complete or partial remission	**OR = 0.3 (0.1–0.7)**	**0.003**	**OR = 0.17 (0.06–0.46)**	**<0.001**	+
	Previous treatment with immunoglobulins	Fernandez-Nebro et al. [31]	113	Complete or partial remission	**OR = 0.3 (0.3–0.7)**	0.007	NS	0.13	+
	Previous treatment with prednisolone ≥ 100 mg/d	Fernandez-Nebro et al. [31]	116	Complete or partial remission	OR = 1.3 (1.115.0)	0.032	**OR = 7.3 (1.6–32.9)**	**0.010**	+

Laboratory biomarker	BCD < 12 vs ≥ 12 mo	Dias et al. [30]	34/64	BILAG score at 6 mo	8.78 vs 5.89	0.004	NP	NP	+
				BILAG score at 12 mo	7.64 vs 5.29	0.017	NP	NP	+
	Complete vs incomplete BCD	Vital et al. [13]	16/21	Major or partial clinical response	**16 (100%) vs 14 (66.7%)**	**0.008**	NP	NP	+
					**RR = 1.5 (1.10–2.02)**				

BCD, B-cell depletion; ES, effect size; CI, confidence interval; NP, information not presented; NS, not significant; RR, relative risk; SLEDAI, systemic lupus erythematosus disease activity.

For overall quality of evidence: +, very low; ++, low; +++, moderate; and ++++, high.

Two other genetic variants—174 IL-6 (rs1800795) and Fc gamma-receptor III a (FCGR3A) 158F/V were evaluated in a single cohort study each and were not related to clinical response [34], [35] (Table 3).

#### Disease-related factors (PICO 2 and 6)

##### Disease phenotype

BILAG-defined mucocutaneous or musculoskeletal system involvement were also assessed as pre-specified subgroups analysis in the EXPLORER trial with no difference in effect estimates found between both interventions groups [8]. The QoE of this RCT was downgraded due to these being subgroup analyses with no tests for interaction and providing exploratory evidence only.

Similarly, the prognostic value of skin phenotype on response and future flare in two cohort studies was unclear with both assessed as having high risk of bias [31], [37] (Table 3, Table 5, Table 6). In a single cohort study, those with previous serious haematologic disorder were 83% less likely to achieve a beneficial response [31] (very low QoE).

**Table 4:** Summary of evidence for factors associated with overall clinical relapse or flare

Factor identified		Study	*N*	Outcome	Unadjusted ES (95% CI)/univariable analysis	*p* Value	Adjusted ES (95% CI)	*p* Value	Overall quality
Laboratory biomarkers	Baseline high (>100 IU/ml) vs low or normal anti-dsDNA antibodies	Lazarus et al. [27]	26/35	Early relapse	NP	NS	NP	NP	+
	Baseline presence vs absence of anti-dsDNA	Catapano et al. [29]	14/17	Time of relapse	NP	NS	NP	NP	+
	Baseline presence of anti-ENA antibodies vs lack of anti-ENA	Ng et al. [33]	21/7	Flare	NP	0.007	**OR = 8.0 (1.2–55)**	**0.034**	+
		Vital et al. [13]	39	Early relapse	NP	Sig	NP	NP	+
		Catapano et al. [29]	10/21	Time of relapse	NP	NS	NP	NP	+
	Baseline BAFF levels	Carter et al. [26]	35	Relapse	NP	>0.55	NP	NP	+
	B-cell repopulation: higher number of memory cell	Vital et al. [13]	32	Early relapse	NP	0.02	NP	NP	+
	B-cell repopulation: number of plasmablasts >0.0008 × 10^9^/l vs <0.0008 × 109/l	Vital et al. [13]	32	Early relapse	80% vs 0%	<0.001	NP	NP	+
				Late relapse	100% vs 27%	NP	NP	NP	+
	B-cell repopulation with higher (>100 IU/ml) vs lower anti-DNA antibodies levels	Lazarus et al. [27]	17/20	Early relapse	NP	0.02	NP	NP	+

Anti-ENA, anti-extractable nuclear antigen; BAFF, B-cell-activating factor; BCD, B-cell depletion; ES, effect size; CI, confidence interval; NP, information not presented; NS, not significant; OR, odds ratio; Sig, significant.

For overall quality of evidence: +, very low; ++, low; +++, moderate; and ++++, high.

**Table 5:** Summary of evidence for factors associated with cutaneous response

Factor identified		Study	*N*	Cutaneous outcome	Unadjusted ES (95% CI)/univariable analysis	*p* Value	Adjusted ES (95% CI)	*p* Value	Overall quality
Disease related	Subtype skin disease: ACLE vs CCLE	Vital et al. [37]	14/8	Response	6 (42.9%) vs 0 (0.0%)	0.14	NP	NP	+
					RR = 7.8 (0.49–122.65)				

Laboratory biomarker	Baseline positive vs negative anti-Ro/SSA antibodies	Vital et al. [37]	16/10	Response	**3 (18.8%) vs 6 (60.0%)**	**0.04**	NP	NP	+
					**RR = 0.31 (0.10–0.97)**				
	Baseline positive vs negative anti-La/SSB antibodies	Vital et al. [37]	8/18	Response	2 (25.0%) vs 7 (38.9%)	0.65	NP	NP	+
					RR = 0.64 (0.17–2.43)				
	Baseline positive vs negative anti-dsDNA antibodies	Vital et al. [37]	14/8	Response	7 (50.0%) vs 2 (16.7%)	0.11	NP	NP	+
					RR = 3.0 (0.82–5.17)				
	Baseline positive vs negative anti-Sm antibodies	Vital et al. [37]	4/22	Response	1 (25.0%) vs 8 (36.4%)	0.68	NP	NP	+
					RR = 0.68 (0.62–2.17)				
	Baseline positive vs negative anti-RNP antibodies	Vital et al. [37]	7/19	Response	0 (0.0%) vs 7 (47.4%)	0.14	NP	NP	+
					RR = 0.13 (0.008–2.0)				
	Low vs normal C3	Vital et al. [37]	9/17	Response	3 (33.3%) vs 6 (35.3%)	0.09	NP	NP	+
					RR = 0.94 (0.30–2.91)				
	Low vs normal C4	Vital et al. [37]	11/16	Response	4 (36.4%) vs 5 (31.3%)	0.78	NP	NP	+
					RR = 1.16 (0.40–3.38)				
	Complete (0.0001 × 10^9^ cells/l) vs incomplete BCD	Vital et al. [37]	10/16	Response	4 (40.0%) vs 5 (31.3%)	0.64	NP	NP	+
					RR = 1.28 (0.44–3.66)				

ACLE, acute cutaneous lupus erythematosus; BCD, B-cell depletion; CCLE, chronic cutaneous lupus erythematosus; ES, effect size; NP, information not presented; NS, not significant; SCLE, subacute cutaneous lupus erythematosus; SLEDAI, systemic lupus erythematosus disease activity.

For overall quality of evidence: +, very low; ++, low; +++, moderate; and ++++, high.

**Table 6:** Summary of evidence for factors associated with cutaneous relapses

Factor identified		Study	*N*	Cutaneous outcome	Unadjusted ES (95% CI)/univariable analysis	*p* Value	Adjusted ES (95% CI)	*p* Value	Overall quality
Disease related	Subtype skin disease: ACLE vs CCLE	Vital et al. [37]	14/8	Relapse	6 (42.9%) vs 0 (0.0%)	0.14	NP	NP	+
					RR = 7.8 (0.49–122.65)				

Laboratory biomarker	Baseline anti-Ro/SSA antibodies positive vs negative	Vital et al. [37]	17/15	Relapse	8 (47.1%) vs 5 (33.3%)	0.44	NP	NP	+
					RR = 1.41 (0.58–3.38)				
	Baseline positive vs negative anti-La/SSB antibodies	Vital et al. [37]	9/23	Relapse	4 (44.4%) vs 8 (34.8%)	0.60	NP	NP	+
					RR = 1.28 (0.51–3.20)				
	Baseline positive vs negative anti-dsDNA antibodies	Vital et al. [37]	17/15	Relapse	7 (41.2%) vs 5 (33.3%)	0.65	NP	NP	+
					RR = 1.24 (0.49–3.08)				
	Baseline positive vs negative anti-Sm antibodies	Vital et al. [37]	4/28	Relapse	0 (0.0%) vs 12 (42.9%)	1.07	NP	NP	+
					RR = 0.23 (0.01–3.32)				
	Baseline positive vs negative anti-RNP antibodies	Vital et al. [37]	7/25	Relapse	4 (57.1%) vs 8 (32.0%)	0.18	NP	NP	+
					RR = 1.78 (0.75–4.21)				
	Low vs normal C3	Vital et al. [37]	12/20	Relapse	6 (50.0%) vs 6 (30.0%)	0.25	NP	NP	+
					RR = 1.66 (0.69–4.00)				
	Low vs normal C4	Vital et al. [37]	14/18	Relapse	6 (42.9%) vs 6 (33.3%)	0.58	NP	NP	+
					RR = 1.29 (0.53–3.13)				
	Complete (0.0001 × 10^9^) cells/l vs incomplete BCD	Vital et al. [37]	12/20	Relapse	3 (25.0%) vs 9 (45.0%)	0.29	NP	NP	+
					RR = 0.56 (0.19–1.66)				

ACLE, acute cutaneous lupus erythematosus; BCD, B-cell depletion; CCLE, chronic cutaneous lupus erythematosus; ES, effect size; NP, information not presented; NS, not significant; SCLE, subacute cutaneous lupus erythematosus; SLEDAI, systemic lupus erythematosus disease activity.

For overall quality of evidence: +, very low; ++, low; +++, moderate; and ++++, high.

A single (very low QoE) cohort study also found that membranous and proliferative LN had similar renal responses following B-cell depleting (BCD) therapy [32] (Table 7).

**Table 7:** Summary of evidence for factors associated with renal response

Factor identified		Study	*N*	Renal outcome	Unadjusted ES (95% CI)/univariable analysis	*p* Value	Adjusted ES (95% CI)	*p* Value	Overall quality
Disease related	Duration of lupus nephritis, mo	Lindholm et al. [28]	11/6	Complete/partial response vs no response	9 vs 19	NS	NP	NP	+
	MLN class vs PLN class	Jónsdottir et al. [32]	15/28	Increase in serum albumin	NP	NS	NP	NP	+
				Mean serum creatinine levels improved	Only in MLN	NP	NP	NP	+
				Reduction in proteinuria	NP	NS	NP	NP	+
				Improvement in C3	NP	NS	NP	NP	+
				Reduction in anti-dsDNA	Only in PLN	**<0.02**	NP	NP	+

Laboratory biomarker	Baseline serum creatinine, μmoles/l	Lindholm et al. [28]	11/6	Complete/partial response vs no response	86.1 ± 30.9 vs 207.2 ± 86.6	0.006	NP	NP	+
	Baseline eGFR ≥ 30 vs < 30 ml/min	Lindholm et al. [28]	13/4	Complete/partial response	11 (84.6%) vs 0 (0.0%)	0.58	NP	NP	+
					RR = 8.21 (0.58–115.21)				
	Baseline proteinuria, g/24 h	Lindholm et al. [28]	11/6	Complete/partial response vs no response	3.4 ± 2.1 vs 5.0 ± 1.6	NS	NP	NP	+
	Baseline anti-dsDNA antibodies, U/ml	Lindholm et al. [28]	11/6	Complete/partial response vs no response	38 ± 4.9 vs 37.5 ± 8.0	NS	NP	NP	+
	Baseline complement C3, g/l	Lindholm et al. [28]	11/6	Complete/partial response vs no response	1.0 ± 0.1 vs 1.1 ± 0.3	NS	NP	NP	+
	Baseline detectable CD 19+ lymphocyte	Lindholm et al. [28]	11/6	Complete/partial response vs no response	9 (81.8%) vs 2 (33.3%) RR = 2.45 (0.76–7.87)	NS	NP	NP	+

BCD, B-cell depletion; CCLE, chronic cutaneous lupus erythematosus; eGFR, estimated glomerular filtration rate; ES, effect size; HCQ, hydroxychloroquine; MLN, membranous lupus nephritis; NP, information not presented, NS, not significant; PLN, proliferative lupus nephritis.

For overall quality of evidence: +, very low; ++, low; +++, moderate; and ++++, high.

##### Disease severity

A single RCT found that baseline BILAG A score did not predict a differential clinical response to RTX compared to control at week 52 [8]. In contrast, high baseline SLEDAI score was associated with better RTX global response at 6 months in one cohort study with high risk of bias [31] (Table 3).

A longer duration (median 19 months) of lupus nephritis (LN) was associated with a lower likelihood of renal response in one cohort with serious limitations [28]. The age-adjusted Charlson comorbidity index and the number of severely affected organ systems were associated with more severe adverse events and more severe infections with RTX therapy in one cohort study (very low QoE) (Table 8) [31].

**Table 8:** Summary of evidence for factors associated with side effects

Factor identified		Study	*N*	Overall outcome	Unadjusted ES (95% CI)/univariable analysis	*p* Value	Adjusted ES (95% CI)	*p* Value	Overall quality
Disease related	Comorbidity*	Fernandez-Nebro et al. [31]	125	Adverse events	**HR = 1.6 (1.1–2.4)**	**0.030**	**HR = 1.6 (1.0–2.6)**	**0.049**	+
	No. of severely involved organ systems (per organ involved)	Fernandez-Nebro et al.[31]	125	Adverse events	**HR = 2.0 (1.4–2.9)**	**<0.001**	**HR = 2.0 (1.3–2.9)**	**0.001**	++
	Previous treatment with steroid bolus (yes/no)	Fernandez-Nebro et al. [31]	125	Adverse events	**HR = 5.4 (2.0–14.8)**	**0.001**	**HR = 5.9 (1.9–18.4)**	**0.002**	++

Laboratory biomarker	Baseline high leucocyte count, ×10^9^/l	Fernandez-Nebro et al. [31]	125	Adverse events	**HR = 1.2 (1.0–1.3)**	**0.045**	**HR = 1.2 (1.0–1.4)**	**0.046**	+
	Complete BCD depletion vs incomplete BCD cell depletion	Vital et al. [13]	16/21	Hospital admissions	4 (25%) vs 10 (47.6%)	NP	NP	NP	+
					RR = 0.54 (0.20–1.37)				

ES, effect size; HR, hazard ratio; NP, information not presented; NS, not significant; SLEDAI, systemic lupus erythematosus disease activity; TC, total cholesterol; TGs, triglycerides.

For overall quality of evidence: +, very low; ++, low; +++, moderate; and ++++, high.

⁎ Age-adjusted Charlson comorbidity index.

The role of previous treatments, a potential proxy of more severe disease or a different phenotype, has been evaluated in only two studies (one RCT and one cohort study). In the EXPLORER trial, a post hoc analysis of patients in previous treatment with methotrexate found a greater fall in mean BILAG global scores in RTX vs placebo-treated patients. There was however no difference in achievement of the primary end point (low QoE) [8]. Previous treatment with prednisone >100 mg/d was related to better clinical response (very low QoE) and previous treatment with steroid bolus was associated with more adverse events (low QoE) in one cohort study. It was unclear what other factors were adjusted for in this cohort [31]. A univariable analysis in the same cohort also showed that previous treatment with immunoglobulins was associated with a reduced likelihood of clinical response; an association not confirmed in a multivariate analysis [31] (Table 3).

#### Laboratory biomarker values (PICO 3 and 7)

##### Baseline laboratory biomarkers

In a post hoc subgroup analysis from the EXPLORER trial that used normalisation of serology as an end point, patients with positive dsDNA (>30 IU/ml) who lacked RNA-binding protein (RBP) had reduced anti-dsDNA antibodies after RTX treatment compared to placebo-treated patients. In contrast, patients with both dsDNA and RBP (>120 AU/ml) antibodies had a similar reduction in anti-dsDNA antibodies in both treatment arms [25]. This study also found that repopulation of CD19+ B cells in dsDNA+RBP+ and dsDNA+RBP− patients were similar despite differences in anti-dsDNA antibodies levels (Table 9). The QoE of this RCT was downgraded due to unclear allocation concealment and post hoc subgroup analysis with insufficient evidence.

**Table 9:** Summary of evidence for factors associated with changes in biomarkers

Factor identified		Study	*N*	Overall outcome	Unadjusted ES (95% CI)/univariable analysis	*p* Value	Adjusted ES (95% CI)	*p* Value	Overall quality
Laboratory biomarker values	Baseline high anti-dsDNA (>123/ml)	Rovin et al. [9]	72	B-cell depletion	NP	**Sig**	NP	NP	+
	Baseline anti-dsDNA titres	Vital et al. [13]	37	Incomplete B-cell depletion	NP	NS	NP	NP	+
	Baseline anti-dsDNA^+^RNP^−^ vs anti-dsDNA+RNP^+^	Tew et al. [25]	97/68	Decreased anti-dsDNA	NP	**<0.025**	NP	NP	+
				Increased complement	NP	NS	NP	NP	+
	Baseline anti-ENA presence	Vita et al. [13]	37	Incomplete B-cell depletion	NP	NS	NP	NP	+
	Baseline low C3 or C4 levels	Vital et al. [13]	37	Incomplete B-cell depletion	NP	NS	NP	NP	+
	Baseline median levels of memory, cells/l	Vital et al. [13]	37	Complete depletion vs persistent B cells	0.0065 × 10^9^ vs 0.0157 × 10^9^	**0.049**	NP	NP	+
	Baseline median levels of plasmablast, cells/l	Vital et al. [13]	37	Complete depletion vs persistent B cells	0.0015 × 10^9^ vs 0.0037 × 10^9^	**0.030**	NP	NP	+
	Baseline high vs low BAFF	Tew et al. [25]	16/9	Changes in anti-dsDNA and complement	NP	**Sig**	NP	NP	+
	Baseline BAFF levels, ng/ml	Carter et al. [26]	34	Time to peripheral B-cell repopulation <26 wk vs >26 wk	1.12 ± 0.20 vs 1.52 ± 0.38	**>0.05**	NP	NP	+
	Baseline positive vs negative IFN signature	Tew et al. [25]	16/9	Changes in anti-dsDNA and complement	NP	NS	NP	NP	+

BAFF, B-cell-activating factor; ES, effect size; HR, hazard ratio; NP, information not presented; NS, not significant; Sig, significant; SLEDAI, systemic lupus erythematosus disease activity; TC, total cholesterol; TGs, triglycerides.

For overall quality of evidence: +, very low; ++, low; +++, moderate; and ++++, high.

Patients with baseline renal impairment (high-serum creatinine levels and a GFR < 30 ml/min) were less likely to have a renal response whilst a higher baseline proteinuria was not associated with renal responses in one cohort study. Analyses were not adjusted for potential confounding [28] (Table 7). Baseline high leucocyte count was found to be related to an increased risk of severe infections during RTX in one cohort study [31] (very low QoE) (Table 8).

A number of studies assessed baseline levels of biomarkers including anti-dsDNA antibodies, low C3, and/or C4 complement, serum B-cell-activating factor (BAFF) and baseline CD19 counts. These studies were rated as low QoE and did not find any of these to be predictors of treatment outcome. Whilst four cohort studies assessed anti-DNA against a range of global- and organ-specific response measures, these other baseline biomarkers have only been assessed in one or two studies each.

Univariable analysis from two cohorts [33] found an association between anti-extractable nuclear antigen antibody (anti-ENA) and flares, but another cohort failed to find this association and only one study confirmed this in a multivariable analysis [33]. Evidence from these studies was downgraded because of inconsistency and high risk of bias.

Regarding specific anti-ENA antibody specificities, anti-Ro was associated with poorer mucocutaneous responses but not with mucocutaneous flares in univariable analysis from one small cohort [37] (*n* = 26) very low QoE) (Table 5, Table 6).

##### Pharmacodynamic biomarkers post-RTX

Two cohorts assessed the association between the degree of BCD and clinical response (one on global response and one for mucocutaneous response). One cohort study (*n* = 37) found that all patients with complete B-cell depletion at 6 weeks (*n* = 16) had higher major or partial global clinical responses at 26 weeks, and all non-responders had persistent B cells after RTX therapy (*n* = 14) [13]. No association between the degree of initial B-cell depletion and response was observed for cutaneous disease (*n* = 26) [37] (Table 3, Table 4), and no association was found when using relapse as the outcome in this subgroup.

One cohort study examined whether BCD could have a relationship with harm during RTX therapy (rates of hospitalisation). There was no adverse influence on safety in patients with complete BCD or prolonged suppression of memory and plasma cell numbers [13] (Table 8).

The duration of BCD was evaluated in one cohort study (*n* = 98), with longer duration (≥12 months) of depletion being associated with a better outcome at 6 and 12 months. Similarly, lymphopenia at any time during the course of the patient׳s disease course was also associated with a better outcome (longer duration of depletion) [30] (Table 3). The quality of evidence for the association of the degree or duration of BCD with treatment outcomes or harm was graded as very low given the imprecision of estimates and limitations of study design.

With regard to peripheral B-cell repopulation, memory cells and plasmablasts repopulation at 26 weeks was significantly associated with earlier relapse and there were also significantly higher numbers of memory cells and plasmablasts (≥0.0008 × 10^9^/l) in patients with earlier relapse in one cohort study (very low QoE) (Table 4).

In another cohort (very low QoE), higher B-cell numbers could be observed as early as 8 weeks post-RTX in those with early (before 18 months) relapse. Also, in this study early relapse was associated with lower levels of repopulation in patients with high (levels of anti-dsDNA antibodies >100 IU/ml) [27] (Table 4).

## Discussion

This systematic review aimed to identify potential predictors of differential treatment effect (moderators) in RCTs and prognostic factors from cohort studies for outcomes of RTX therapy for SLE. Using validated tools, the overall quality of most of the investigated predictive or prognostic factors was low or very low, which means that our confidence in the majority of these is very limited. The quality of evidence was affected mainly by limitations associated with (1) the evidence arising generally from explanatory studies conducted in an early, hypothesis-generating phase of investigation; (2) risk of publication bias (or small study) due to associations of prognostic factors with outcome mainly reported by a very small number of studies with small sample size; (3) several included studies only reported “*p*” values rather than effect sizes that limits conclusions to be drawn about the precision of effect estimates and clinical relevance of the study findings; (4) results derived from subgroup analyses without interaction tests in only a small number of studies; (5) many associations explored in only one study; and (6) several studies reported from the same centre or cohort, which makes evaluating consistency of results across studies difficult. Even when assessing a common marker such as anti-ENA antibodies and B-cell dynamics post-RTX different definitions and cut-points between studies limit the ability to validate or confirm findings. Further primary hypothesis-driven research based on longitudinal studies of sufficient sample size exploring factors associated with RTX outcomes in SLE patients is therefore needed to confirm and validate which, if any of these, factors truly predict the outcomes of RTX therapy.

A number of factors associated with clinical response to rituximab are suggested by this review that will require further study. In a pre-specified secondary analysis of the EXPLORER trial, African American/Hispanic patients who received RTX were more likely to achieve the primary end point compared to the same race/ethnicity groups receiving standard of care (control arm). This difference was however driven by a lower response to standard of care in African American/Hispanic patients compared to overall trial population [8]. It therefore remains to be seen whether this simply represents overall less stable disease in this subgroup or whether RTX has indeed a differential effect in these race/ethnicity subgroups. Similarly in the same trial, RTX-treated patients receiving co-therapy with MTX achieved a lower global BILAG score by 52 weeks compared to placebo-treated patients; however, the primary end point of the trial did not differ according to co-therapy. Whether this represents a particular synergy between RTX and MTX in SLE patients or not cannot be deduced from this data.

In the cohort studies, the majority of prognostic factors were limited to small early-phase studies and often a single study examining each factor. Several factors including anti-dsDNA status were examined across a number of cohorts [27], [28], [29], [37] and did not show any association with RTX responses (either clinical or serological changes). Any conclusions are again limited since most cohorts were small and used different outcomes measures and also there have been a number of cohorts from which multiple publications have arisen and it is unclear about the degree of overlap in the patients studied. We therefore cannot confidently exclude such a factor as being of importance.

A number of factors suggested in at least one study included IL2/IL21 SNP GG genotype and G allele, which were associated with global clinical response in one cohort study [36]. Whilst this needs confirmation in a much larger cohort, attention also needs to be paid to whether it is a predictor of response only for RTX or whether it simply predicts a better response to other immune-modulatory therapy as well. This will be important to determine the true significance of such a predictive biomarker. Of interest in a recent systematic review of predictors of response to biologic therapy in RA, conflicting results were reported for FCGR variants and no association between RTX responses and IL-6 promoter 174 were observed [38]. In SLE, these variants also did not show any association with global SLE responses to RTX.

Two single-centre cohort studies found that the level of B-cell depletion achieved as well as the early return of B cell (especially plasmablasts and memory B cells) were associated with early relapses in studies from two cohorts [13], [27]. Such factors are of interest as the latter pattern of B-cell depletion and return are likely to be specific to the action of RTX and therefore will act as a pharmacodynamic measure of RTX efficacy and future relapses. At a pragmatic and clinical level, more complex monitoring than simply assessing baseline factors may therefore be needed to fully understand the specificity of responses to RTX and to plan more tailored therapy. Such dynamic markers of course have the limitation of not allowing clinicians to decide *a priori* whether to treat or not treat with RTX but will be of great value in planning future courses and potentially helping to make an early declaration of therapeutic “failure” so that other therapies can be initiated sooner. For such factors to be validated to the degree of certainty required to formalise therapeutic decisions will however require large multicentre, quality-assured confirmatory studies. Such studies do also provide potentially important insights into the mechanisms of action of RTX in mediating prolonged responses in some patients. Recent work on regulatory B cells suggests that it is likely to be the pattern of return of B-cell subsets rather than total B-cell numbers that determine future relapses, providing a potential explanation for the inconsistencies in the value of B-cell markers in determining response in the cohorts reviewed [39].

We noted that there are differences across studies in how specific organ systems responded to RTX therapy. For example, one small study suggested that Ro+ve patients and those with chronic cutaneous lupus had poorer responses [37]. In addition, patients with elevated creatinine and those with longer duration of LN responded less well in one cohort study [28]. Whether these observations relate to more scarring/fibrosis and hence a lower likelihood of response in these subsets or whether they point to a different pathogenesis (e.g., T-cell or IFN-driven disease) will require further study. All these studies were however rated as low or very low QoE and therefore confirmation of these observations are necessary in parallel with a better understanding of the explanations for any such lack of response to RTX.

Factors related with drug administration such as concomitant therapy, administration schedules, or human antichimeric antibodies (HACA) were not included in our analysis as such studies are more focused on optimal delivery of the treatment, rather than investigating which patient or disease characteristics (prognostic factors) are associated with positive outcomes of treatment.

The strength of this review lies in the fact that this is the first comprehensive review that provides a summary of the possible predictive or prognostic factors in SLE patients with RTX therapy. However, we recognise several limitations to the review. First, due to the small number of studies included and the heterogeneity between studies we were not able to combine the results of studies from a quantitative perspective. We have also synthesised the evidence on associations between a single potential prognostic factor and an outcome variable when the study employed either univariable analyses or multivariable analyses. By including both types of analyses we may have introduced a degree of heterogeneity. One major limitation is that even in these best case scenarios, the variables controlled for in each analysis may differ between studies and we therefore cannot assume that any of the single-identified associations would remain if more consistent modelling was applied in all studies. Second, most studies included in this systematic review were in SLE patients who failed to respond to conventional treatment, therefore, we cannot extrapolate our results to patients with newly diagnosed SLE, who are candidate for standard therapy. A few small studies have assessed the effectiveness of rituximab in those patients [40], [41], however, some of these studies were not included in our review due to mainly either no prognostic factor analysis was provided or less than 30 patients were included (Supplementary File A, Table A.7). Third, it is also possible that we have missed studies that are not indexed in these databases, but by checking references of included studies, we made every effort to identify all relevant articles. Finally, no attempt was made to contact authors to obtain individual patient-level data or carry out a comprehensive meta-analysis.

Clinical experience, observational studies, and several international guidelines [10], [11] support the use of RTX for the treatment of refractory SLE as well as disease with sustained activity that persists despite conventional immunosuppression therapy. Despite the more widespread use and studies supporting its efficacy, we found limited evidence to predict which patient groups will respond better (or worse) to RTX to permit a stratified approach to the use of this agent. A number of demographic, serological, genetic, and pharmacodynamic markers were identified in this review; however, most studies addressing these prognostic factors were hypothesis generating and therefore cannot be used to make any specific recommendations for routine clinical practice. It is therefore important to validate any predictive or prognostic factors in hypothesis-testing studies and determine whether such markers are associated with SLE outcomes in general or whether they are specific for RTX therapy. Such an approach will pave the way for more personalised use of this agent in the future.
