# Supplementary material for: Predictive and prognostic factors influencing outcomes of rituximab therapy in systemic lupus erythematosus (SLE): A systematic review
Source: Semin Arthritis Rheum. 2017 Dec;47(3):384–96. doi: 10.1016/j.semarthrit.2017.04.010 (PMC5695978; doi:10.1016/j.semarthrit.2017.04.010)
Supplement: Supplementary file 1 — Supplementary material [file mmc1.docx]

## Supplementary file A

**Table A.1 Search strategy**

| 1. exp Lupus Erythematosus, Systemic/ 2. systemic lupus erythematosus.mp. 3. exp Lupus Nephritis/ 4. lupus nephritis.mp. 5. 1 or 2 or 3 or 4 6. exp rituximab/ 7. rituximab.mp. 8. anti-CD20.mp. 9. 6 or 7 or 8 10. exp Prognosis/ 11. exp Treatment Outcome/ 12. 10 or 11 13. 5 and 9 and 12   Search Strategy for MEDLINE (Ovid) adapted for other relevant databases and searches. |
| --- |

Exp= explode function searches for an index term (subject heading); mp= multiple purpose (title, abstract, original title, name of substance word, subject heading word, keyword heading word, protocol supplementary concept word, rare disease supplementary concept word, unique identifier).

**Table A.2 Definitions according with individual studies included**

| **Author** | **Factor or predictor associated with outcome** | **Definition** | **Outcome** | **Definition** |
| --- | --- | --- | --- | --- |
| Catapano 2010 | Baseline anti-dsDNA and anti-ENA | Not defined | Clinical relapse | An increase in disease activity which required an increase in the prednisolone dose |
| Carter 2013 | Baseline and after BCD serum BAFF levels | Human BAFF Quantikine ELISA | Clinical Relapse | 1 new A grade or 2 new B grades for any organ system |
|  |  |  | Levels IgG and IgA | Not indicated |
|  |  |  | Anti-dsDNA antibody levels | ELISA |
| Dias 2015 | Longer duration of B cell depletion | Patients who repopulated after 12 months  B cell depletion: 0.03-0.40 x10^9^/l until 2010 and 0.69-0.11x10^9^/l after 2010. | Clinical improvement | A decrease in BILAG numerical score. |
| Fernandez-Nebro 2012 | Previous treatment with immunoglobulins | No definition indicated | Clinical response | SELENA-SLEDAI score ≤ two and a SELENA-SLEDAI Flare Index of zero |
|  | High dose of prednisone | History of treatment with daily doses above 100 mg/day to control of the lupus activity |  |  |
|  | Previous discoid rash | Not definition indicated |  |  |
|  | Severe hematologic disorder | Not definition indicated |  |  |
|  | Baseline activity | SELENA-SLEDAI |  |  |
|  | Comorbidity | Age-adjusted Charlson comorbidity index | Adverse events | Medical Dictionary for Regulatory Activities Terminology |
|  | No. of severely involved organ systems | Severe, when two or more of these organs had been affected or aggressive therapy had been required for the complications, such as more than 50 mg of prednisone daily, pulse therapy with steroids or cyclophosphamide boluses |  |  |
|  | Baseline leukocyte count, x10^9^/L | Not definition indicated for high count |  |  |
|  | Previous treatment with steroid bolus | Not definition indicated |  |  |
| Jónsdóttir 2010 | Histopathological | Membranous (WHO Class V) vs Proliferative (WHO Class III or IV) LN | Renal improvement | Reduction in proteinuria and an increase in serum albumin  Reduction in anti-dsDNA titre and improvements in complement C3 levels |
| Lazarus 2012 | Baseline and after BCD anti-dsDNA antibody levels | ELISA (normal <50 U/ml), High anti-DNA levels: >100 IU/l | Clinical relapse | An increase in the clinical indices of active disease, based on the classic BILAG index.  Early relapse: before 18 months |
|  | Changes in anti-dsDNA antibody levels | Percentage change in anti-dsDNA antibody levels |  |  |
|  | Baseline B cells | CD19+ B cells, flow cytometer |  |  |
|  | B cell repopulation | CD19+ B cells, >0.01x10^9^ B cells/l |  |  |
|  | Different B-cell phenotypes | Plasmablast and double negative memory B cells |  |  |
| Lindholm 2008 | LN duration | Longer duration of nephritis | Renal response | Normal SCr and serum albumin levels, inactive urinary sediment, and a 24-hour urinary albumin secretion < 0.5 g; a partial response was defined as ≥ 50% improvement of the renal measures being abnormal at baseline and absence of deterioration of any of them. |
|  | Baseline eGFR | eGFR > 30 ml/min |  |  |
|  | Baseline serum creatinine | Higher serum creatinine levels |  |  |
|  | Baseline proteinuria | Urine albumin loss, g/24 h |  |  |
|  | Baseline Anti-dsDNA | Radioimmunoassay and also by indirect immunocytochemistry using Crithidia lucillae |  |  |
|  | Baseline detectable B cell | Not defined |  |  |
| Marquez 2013 | Genetic factors | rs6822844 G/T polymorphism at the IL2–IL21 region was analysed by TaqMan assay | Clinical response | Complete response was defined as disappearance of all symptoms and signs of the systemic disease that recommended the use of rituximab. Partial response was defined as a significant improvement (at least 50 %) of initial disease activity, based on clinical judgment. Responders included complete responders and partial responders |
| Merrill 2010 | Age | ≤40 years or >40 years | Major clinical response  Partial clinical response  No clinical response | BILAG C scores or better in all organs at week 24 without experiencing a severe flare (1 new domain with a BILAG A score or 2 new domains with a BILAG B score) from day 1 to week 24, and maintaining this response without a moderate or severe flare (≥1 new domains with a BILAG A or B score) to week 52 |
|  | Gender | Female or male |  |  |
|  | Race | African American/Hispanic versus others |  |  |
|  | Assigned prednisone dose | 0.5 or 0.75 or 1.0 mg/kg/day |  | 1) achieving BILAG C scores or better at week 24 and maintaining this response without a new BILAG A or B score for 16 consecutive weeks, 2) achieving no more than 1 organ with a BILAG B score at week 24 without achieving ≥1 new BILAG A or B score to week 52, or 3) achieving a maximum of 2 BILAG B scores at week 24 without developing BILAG A or B scores in new domains until week 52 if the baseline BILAG score for the patient was 1 A score plus ≥2 B scores, ≥2 A scores, or ≥4 B scores |
|  | Background immunosuppressant | Azathioprine or methotrexate or mycophenolate mofetil |  |  |
|  | Duration of lupus | Years |  |  |
|  | Baseline BILAG A | Score |  |  |
|  | Baseline BILAG-defined mucocutaneous or musculoskeletal system involvement | BILAG A or BILAG B |  | Failure to meet the definition of a major clinical response or a partial clinical response. Patients who terminated the study early were scored as having no clinical response |
| Ng 2007 | Anti-ENA | ELISA | Clinical flare | A new ‘A’ or a new ‘B’ present on two consecutive occasions in any organ system of the BILAG activity index. |
| Robledo 2012 | Genetic factors | - 174 IL-6 polymorphism | Clinical response | ACR and EULAR recommendations |
| Robledo 2012 | Genetic factors | FCGR3A-158F/V polymorphism | Clinical response | ACR and EULAR recommendations |
| Rovin 2012 | B cells at baseline and B cells depletion | Not indicated | Overall response | Criteria for a CRR included the following: normal SCr level if it was abnormal at baseline, or a SCr level of ≤ 115% of baseline if it was normal at baseline; inactive urinary sediment (<5 RBCs/hpf and absence of RBC casts); and UPC ratio <0.5.  Patients who achieved PR, but not CRR, met the following criteria: SCr level ≤115% of baseline; RBCs/hpf ≤50% above baseline and no RBC casts; and at least a 50% decrease in the UPC ratio to <1.0 (if the baseline UP/Cr was ≤ 3.0) or to ≤3.0 (if the baseline UP/Cr was >3.0) |
| Tew 2010 | Anti-dsDNA | Bindazyme anti-dsDNA EIA kit | Normalization of complement and anti-dsDNA | Immunonephelometry and ELISA |
|  | Anti- anti-RBP (RNP, Sm, SSA (Ro), SSB (La) | AtheNA Multi-Lyte ANA Test System multiplex ELISA kit |  |  |
|  | B cells levels | Four-color flow-cytometry analysis |  |  |
|  | High BAFF at baseline and after BCD | The top 78th percentile of patients (cut-off of 6007 RU) measured by ELISA |  |  |
|  | Interferon signature at baseline and after BCD | Quantitative PCR assay, yielding an interferon signature metric from the interferon signature |  |  |
| Vital 2011 | Baseline anti-ENA | Not defined | Clinical response | Major clinical response no domain rated BILAG A or B at week 26 and no A or B flare between weeks 0 and 26; partial clinical response = maximum of 1 domain with a persistent B rating at 26 weeks with improvement in all other domains rated A or B at baseline, no new grade A flare between weeks 0 and 26, and no new grade B flare in more than 1 single domain between weeks 0 and 26; and nonresponse = patients not meeting the criteria for major clinical response or partial clinical response |
|  | B cell depletion | By flow cytometry, B cell count after rituximab infusion |  |  |
|  | B cell repopulation | Plasmablast level of ≥0.0008 10^9^/liter | Relapse | A new BILAG grade A flare or 2 grade B flares following major clinical response or partial clinical response at 26 weeks (early) or at 52 weeks (later) |
| Vital 2015 | Cutaneous phenotype: ACLE, SCLE and CCLE | The Gilliam classification terminology | Mucocutaneos response    Mucocutaneous flare | Mucocutaneous response and flare were classified according to BILAG mucocutaneous domain score, change in morphology, and change in therapy.  Response: BILAG mucocutaneous: reduction of a BILAG score of A to a score of B, C, or D; reduction of a BILAG score of B to a score of C or D; or reduction of a BILAG score of C (mild mucosal ulceration, mild alopecia, or chilblains) to a score of D with no new topical or immunosuppressive therapy for skin disease  Flare: 1) new mucocutaneous disease with a BILAG score of A–C in a patient with no cutaneous involvement at the time of rituximab treatment, or 2) a new cutaneous lupus morphology in a patient with or without other mucocutaneous lupus at baseline |
|  | Autoantibodies and complement:  anti-dsDNA,  anti-Ro/SSA  anti-La/SSB  anti-Sm  anti-RNP  low C3 and C4 | Not defined |  |  |
|  | Complete B cell depletion | B cells 0.0001 x 10^9^ cells/liter |  |  |

**Abbreviations:** ACLE: acute cutaneous lupus erythematosus; ACR: American College of Rheumatology; BAFF, B-cell–activating factor; anti-ENA: anti-extractable nuclear antigen; BCD: B cell depletion; BILAG: British Isles Lupus Assessment Group index; CCLE: chronic cutaneous lupus erythematosus; CCR: complete renal response; eGFR ; estimated glomerular filtration; EIA: enzyme immunoassay; ELISA: enzyme-linked immunosorbent assay; HACA: human antichimeric antibody; hpf: high-power field; HCQ: hydroxychloroquine; ISN/RPS: International Society of Nephrology /Renal Pathology Society classification; LupusQol index: Lupus Quality of Life; LN: lupus nephritis; PRR: partial renal response; RBCs: red blood cells; SCLE: subacute cutaneous lupus erythematosus; SCr: serum creatinine; SELENA-SLEDAI: Safety of Estrogens in Lupus Erythematosus National Assessment-SLE disease activity index; SNP: single nucleotide polymorphism; Upr/Ucr: urinary protein to urinary creatinine; WHO: World Health Organization classification.

**Table A.3 Quality in Prognostic Studies (QUIPS) tool (Hayden 2013)**

| **Domains** | | **Prompting items for Consideration** | **Ratings** |
| --- | --- | --- | --- |
| **Study Participation**  The study sample adequately represents the population of interest | 1. Adequate participation in the study by eligible persons 2. Description of the source population or population of interest 3. Description of the baseline study sample 4. Adequate description of the period and place of recruitment 5. Adequate description of inclusion and exclusion criteria | | **High bias:** The relationship between the PF and outcome is very likely to be different for participants and eligible nonparticipants  **Moderate bias:** The relationship between the PF and outcome may be different for participants and eligible nonparticipants  **Low bias:** The relationship between the PF and outcome is unlikely to be different for participants and eligible nonparticipants |
| **Study Attrition**  The study data available (i.e., participants not lost to follow-up) adequately represent the study sample | 1. Adequate response rate for study participants 2. Description of attempts to collect information on participants who dropped out 3. Reasons for loss to follow-up are provided 4. Adequate description of participants lost to follow-up 5. There are no important differences between participants who completed the study and those who did not | | **High bias:** The relationship between the PF and outcome is very likely to be different for completing and non-completing participants  **Moderate bias:** The relationship between the PF and outcome may be different for completing and non-completing participants  **Low bias:** The relationship between the PF and outcome is unlikely to be different for completing and non-completing participants |
| **Prognostic Factor (PF) Measurement**  The PF is measured in a similar way for all participants | | 1. A clear definition or description of the PF is provided 2. Method of PF measurement is adequately valid an reliable 3. Continuous variables are reported or appropriate cut points are used 4. The method and setting of measurement of PF is the same for all study participants 5. Adequate proportion of the study sample has complete data for the PF 6. Appropriate methods of imputation are used for missing PF data | **High bias:** The measurement of the PF is very likely to be different for different levels of the outcome of interest  **Moderate bias:** The measurement of the PF may be different for different levels of the outcome of interest  **Low bias:** The measurement of the PF is unlikely to be different for different levels of the outcome of interest |
| **Outcome Measurement**  The outcome of interest is measured in a similar way for all participants | | 1. A clear definition of the outcome is provided 2. Method of outcome measurement used is adequately valid and reliable 3. The method and setting of outcome measurement is the same for all study participants | **High bias:** The measurement of the outcome is very likely to be different related to the baseline level of the PF  **Moderate bias:** The measurement of the outcome may be different related to the baseline level of the PF  **Low bias:** The measurement of the outcome is unlikely to be different related to the baseline level of the PF |
| **Study Confounding**  Important potential confounding factors are appropriately accounted for | | 1. All important confounders are measured 2. Clear definitions of the important confounders measured are provided 3. Measurement of all important confounders is adequately valid and reliable 4. The method and setting of confounding measurement are the same for all study participants 5. Appropriate methods are used if imputation is used for missing confounder data 6. Important potential confounders are accounted for in the study design 7. Important potential confounders are accounted for in the analysis | **High bias:** The observed effect of the PF on the outcome is very likely to be distorted by another factor related to PF and outcome  **Moderate bias:** The observed effect of the PF on outcome may be distorted by another factor related to PF and outcome  **Low bias:** The observed effect of the PF on outcome is unlikely to be distorted by another factor related to PF and outcome |
| **Statistical Analysis and Reporting**  The statistical analysis is appropriate, and all primary outcomes are reported | | 1. Sufficient presentation of data to assess the adequacy of the analytic strategy 2. Strategy for model building is appropriate and is based on a conceptual framework or model 3. The selected statistical model is adequate for the design of the study 4. There is no selective reporting of results | **High bias:** The reported results are very likely to be spurious of biased related to analysis or reporting  **Moderate bias:** The reported results may be spurious or biased related to analysis or reporting  **Low bias:** The reported results are unlikely to be spurious or biased related to analysis or reporting |

**Abbreviation:** PF prognostic factor

**Table A.4 PICO (Population, Intervention, Comparator, Outcome) questions**

| **PICO questions on interventions for moderators (predictors of differential effect)** |
| --- |
| 1. In patients with SLE (P), do sociodemographic factors moderate the effect of rituximab (I) on outcome (O) compared with control (C)? 2. In patients with SLE (P) do disease related factors moderate the effect of rituximab (I) on outcome (O) compared with control (C)? 3. In patients with SLE (P) do laboratory biomarker values moderate the effect of rituximab (I) on outcome (O) compared with control (C)? |
| **PICO questions on prognostic factors** |
| 1. In patients with SLE on rituximab (P), what is the association of sociodemographic factors (I) with outcome (O)? 2. In patients with SLE on rituximab (P), what is the association of genetic factors (I) with outcome (O)? 3. In patients with SLE on rituximab (P), what is the association of disease related factors (I) with outcome (O)? 4. In patients with SLE on rituximab (P), what is the association of laboratory biomarker values (I) with outcome (O)? |

**Table A.5 The GRADE process rating the quality of evidence for predictors of differential response to treatment with rituximab compared with placebo or other active treatments**

| Rating is modified downward:  Risk of bias; based on the Cochrane Collaboration risk of bias tool. 1. Lack of allocation concealment (those enrolling patients are aware of the group to which the next enrolled patient will be allocated [major problem in ‘pseudo’’ or ‘‘quasi’’ randomized trials with allocation by day of week, birth date, chart number, etc.]); 2. Lack of blinding (patient, care givers, those recording outcomes, those adjudicating outcomes, or data analysts are aware of the arm to which patients are allocated [or the medication currently being received in a crossover trial]); 3. Incomplete accounting of patients and outcome events (loss to follow-up and failure to adhere to the intention-to-treat principle in superiority trials; or in noninferiority trials, loss to follow-up, and failure to conduct both analyses considering only those who adhered to treatment, and all patients for whom outcome data are available); 4. Selective outcome reporting bias (incomplete or absent reporting of some outcomes and not others on the basis of the results; 5. Other limitations (stopping early for benefit, use of unvalidated outcome measures [e.g., patient-reported outcomes], carryover effects in crossover trial, recruitment bias in cluster-randomized trials and not clear sponsorship or funded by pharmaceutical industry.  Insufficient evidence for subgroup analysis based on Pincus criteria  Inconsistency; variations in effect estimates across studies with points of effect on either side of the line of no effect, and confidence intervals showing minimal overlap.  Indirectness; differences in population, intervention, outcome and comparison group. Imprecision; number of participants included in the systematic review is appropriate through sample size estimation (if the total number of patients included in a systematic review is less than the number of patients generated by a conventional sample size calculation for a single adequately powered trial [“optimal information size’’]) and confidence interval do include null effect and do include appreciable harm or benefit (i.e. RR under 0.75 or over 1.25).  Publication bias, we did not perform a formal assessment of potential publication bias (small-trial bias), because the review included fewer than 10 trials.  Rating is modified upward:  Large effect: direct evidence, relative risk (RR) =2-5 or RR =0.5-0.2 with no plausible confounders); very large with RR >5 or RR < 0.2 and no serious problems with risk of bias or precision (sufficiently narrow confidence intervals).  Dose response gradient.  All plausible residual confounders or biases would reduce a demonstrated effect, or suggest a spurious effect when results show no effect |
| --- |

**Table A.6** **The GRADE process rating the quality of evidence for prognostic factors in clinical cohorts treated with rituximab**

| Rating is modified downward:  Univariable analysis is applied to identify associations between a number of potential prognostic factors and the outcomes.  Study limitations; moderate or low study quality based on the QUIPS ‘risk of bias’ score.  Inconsistency of results; variations in effect estimates across studies with points of effect on either side of the line of no effect, and confidence intervals showing minimal overlap.  Indirectness of evidence; the study sample does not fully represent the review question defined in the systematic review, but only represents a subset of the population of interest.  Within study imprecision; (1) sample size justification is not provided and there are less than 10 outcome events for each prognostic variable (for dichotomous outcomes) OR there are less than 100 cases reaching endpoint (for continuous outcomes), and (2) no precision in the estimation of the effect size within each primary study, AND - Across study imprecision: there are few studies and small number of participants across studies.  Publication bias likely; publication bias likely when a prognostic factor has been shown to be strong or significant mainly in small cohort studies.  Rating is modified upward:  When moderate or strong associations are reported by most of the primary studies. |
| --- |

**Table A.7 Excluded Full-Text Studies and Reasons for Exclusion**

|  | Study author(s), year of publication & publication title | Reason for exclusion |
| --- | --- | --- |
| 1 | Abud-Mendoza C et al., 2009: Treating severe systemic lupus erythematosus with rituximab. An open study | No prognostic factor analysis |
| 2 | Albert D et al., 2008: Variability in the biological response to anti-CD20 B cell depletion in systemic lupus erythaematosus | 24 patients included |
| 3 | Andrade-Ortega L et al., 2010: Efficacy of rituximab versus cyclophosphamide in lupus patients with severe manifestations. A randomized and multicenter study | No prognostic factor analysis and 19 patients included |
| 4 | Anolik JH et al., 2003: The relationship of FcɤRIIIa genotype to degree of B cell depletion by rituximab in the treatment of systemic lupus erythematosus | 12 patients included |
| 5 | Anolik JH et al., 2004: Rituximab improves peripheral B cell abnormalities in human systemic lupus erythematosus | 18 patients included |
| 6 | Anolik JH et al., 2007: Delayed memory B cell recovery in peripheral blood and lymphoid tissue in systemic lupus erythematosus after B cell depletion therapy | 15 patients included |
| 7 | Arce-Salinas CA et al., 2012: Long-term efficacy of anti-CD20 antibodies in refractory lupus nephritis | 8 patients included |
| 8 | Bang SY et al., 2012: Multicenter retrospective analysis of the effectiveness and safety of rituximab in Korean patients with refractory systemic lupus erythematosus | No prognostic factor analysis |
| 9 | Boletis JN et al., 2009: Rituximab and mycophenolate mofetil for relapsing proliferative lupus nephritis: a long-term prospective study | In combination with MMF |
| 10 | Braun-Moscovici Y et al., 2013: Rituximab: rescue therapy in life-threatening complications or refractory autoimmune diseases: a single center experience | 4 patients included |
| 11 | Cambridge G et al., 2008: B cell depletion therapy in systemic lupus erythaematosus: relationships among serum B lymphocyte stimulator levels, autoantibody profile and clinical response | 25 patients included |
| 12 | Chen H et al., 2011: Low-dose rituximab therapy for refractory thrombocytopenia in patients with systemic lupus erythematosus--a prospective pilot study | 10 patients included |
| 13 | Chiche L et al., 2011: Normalization of serum-free light chains in patients with systemic lupus erythematosus upon rituximab treatment and correlation with biological disease activity | 11 patients included |
| 14 | Condon MB et al., 2013: Prospective observational single-centre cohort study to evaluate the effectiveness of treating lupus nephritis with rituximab and mycophenolate mofetil but no oral steroids | In combination with MMF |
| 15 | Conti F et al., 2011: Rituximab infusion-related adverse event rates are lower in patients with systemic lupus erythematosus than in those with rheumatoid arthritis. | 23 patients included |
| 16 | Dale RC et al., 2014: Utility and safety of rituximab in pediatric autoimmune and inflammatory CNS disease. | 18 patients included |
| 17 | Danes I et al., 2013: Available evidence and outcome of off-label use of rituximab in clinical practice | No prognostic factor analysis |
| 18 | Davies RJ et al., 2013: Rituximab in the treatment of resistant lupus nephritis: therapy failure in rapidly progressive crescentic lupus nephritis | 18 patients included |
| 19 | Diaz-Lagares C et al., 2012: Efficacy of rituximab in 164 patients with biopsy-proven lupus nephritis: Pooled data from European cohorts | Systematic review |
| 20 | El-Hallak M et al., 2007: Clinical effects and safety of rituximab for treatment of refractory pediatric autoimmune diseases | 10 patients included |
| 21 | Ezeonyeji AN et al., 2012: Early treatment with rituximab in newly diagnosed systemic lupus erythematosus patients: a steroid-sparing regimen | Combination therapy and 8 patients included |
| 22 | Fernandez-Nebro A et al., 2014: The effects of rituximab on the lipid profile of patients with active systemic lupus erythematosus: results from a nationwide cohort in Spain (LESIMAB) | No prognostic factor analysis |
| 23 | Galarza-Maldonado C et al., 2008: Should rituximab be considered as the first-choice treatment for severe autoimmune rheumatic diseases? | No prognostic factor analysis |
| 24 | Galarza-Maldonado C et al., 2010:The administration of low doses of rituximab followed by hydroxychloroquine, prednisone and low doses of mycophenolate mofetil is an effective therapy in Latin American patients with active systemic lupus erythematosus | No prognostic factor analysis |
| 25 | Garcia-Carrasco M et al., 2010: Anti-CD20 therapy in patients with refractory systemic lupus erythematosus: a longitudinal analysis of 52 Hispanic patients | No prognostic factor analysis |
| 26 | Gottenberg JE et al., 2005: Tolerance and short term efficacy of rituximab in 43 patients with systemic autoimmune diseases | 13 patients included |
| 27 | Gunnarsson I et al., 2007: Histopathologic and clinical outcome of rituximab treatment in patients with cyclophosphamide-resistant proliferative lupus nephritis | 7 patients included |
| 28 | Harb DM et al., 2014: The off-label use of rituximab for the management of inflammatory disorders: American University of beirut medical center experience | No prognostic factor analysis |
| 29 | Heusele M et al., 2014: Risk factors for severe bacterial infections in patients with systemic autoimmune diseases receiving rituximab | 22 patients included |
| 30 | Hickman RA et al., 2015: The efficacy and safety of rituximab in a chart review study of 15 patients with systemic lupus erythematosus | 15 patients included |
| 31 | Hofman SC et al., 2013: Effects of rituximab-based B-cell depletion therapy on skin manifestations of lupus erythematosus--report of 17 cases and review of the literature | 17 patients included |
| 32 | Iaccarino L et al., 2015: Efficacy and safety of off-label use of rituximab in refractory lupus: data from the Italian Multicentre Registry | No prognostic factor analysis |
| 33 | Jansson AF et al., 2011: B cell depletion for autoimmune diseases in paediatric patients | 19 patients included |
| 34 | Jiang B et al., 2015: Efficacy and safety of rituximab in systemic lupus erythematosus and Sjögren syndrome patients with refractory thrombocytopenia a retrospective study of 21 cases | 21 patients included |
| 35 | Jónsdóttir T et al., 2008: Treatment of refractory SLE with rituximab plus cyclophosphamide: clinical effects, serological changes, and predictors of response | 16 patients included |
| 36 | Jónsdóttir T et al., 2013: Long-term follow-up in lupus nephritis patients treated with rituximab—clinical and histopathological response | Combination therapy and 25 patients included |
| 37 | Lateef A et al., 2010: Use of rituximab in the treatment of refractory systemic lupus erythematosus: Singapore experience | 10 patients included |
| 38 | Leandro MJ et al., 2005: B‑cell depletion in the treatment of patients with systemic lupus erythematosus: a longitudinal analysis of 24 patients | 24 patients included |
| 39 | Lee HT et al., 2014: Increased 8-hydroxy-2'-deoxyguanosine in plasma and decreased mRNA expression of human 8-oxoguanine DNA glycosylase 1, anti-oxidant enzymes, mitochondrial biogenesis-related proteins and glycolytic enzymes in leucocytes in patients with systemic lupus erythematosus | 4 patients included |
| 40 | Lehman TJ et al., 2014: Prolonged improvement of childhood onset systemic lupus erythematosus following systematic administration of rituximab and cyclophosphamide | 12 patients included and combination therapy |
| 41 | Li EK et al., 2009: Is combination rituximab with cyclophosphamide better than rituximab alone in the treatment of lupus nephritis? | No predictor analysis |
| 42 | Looney RJ et al., 2004: B cell depletion as a novel treatment for systemic lupus erythematosus a phase I/II dose-escalation trial of rituximab | 18 patients included |
| 43 | Lu TY et al., 2009: A retrospective seven-year analysis of the use of B cell depletion therapy in systemic lupus erythematosus at university college London hospital: The first fifty patients | No prognostic factor analysis |
| 44 | Machado RIL et al., 2014: Use of rituximab as a treatment for systemic lupus erythematosus: retrospective review | 17 patients included |
| 45 | Marco HS, et al., 2014: The effect of rituximab therapy on immunoglobulin levels in patients with multisystem autoimmune disease | 3 patients included |
| 46 | Marks SD et al., 2005: B lymphocyte depletion therapy in children with refractory systemic lupus erythematosus | 7 patients included |
| 47 | Melander C et al., 2009: Rituximab in severe lupus nephritis: early B-cell depletion affects long-term renal outcome | 20 patients included |
| 48 | Mendoza-Pinto C., 2012: Bone mineral density in systemic lupus erythematosus women one year after rituximab therapy | No prognostic factor analysis |
| 49 | Merrill JT et al., 2011: Assessment of flares in lupus patients enrolled in a phase II/III study of rituximab (EXPLORER) | No predictor analysis |
| 50 | Moroni G et al., 2014: Rituximab vs mycophenolate and vs cyclophosphamide pulses for induction therapy of active lupus nephritis: a clinical observational study | 17 patients included |
| 51 | Olfat M et al., 2015: Rituximab therapy has a rapid and durable response for refractory cytopenia in childhood-onset systemic lupus erythematosus | 24 patients included |
| 52 | Pavon-Sanchez JM, et al., 2013: Rituximab in pediatric patients with systemic lupus erythematosus | 8 patients included |
| 53 | Pepper R et al., 2009: Rituximab is an effective treatment for lupus nephritis and allows a reduction in maintenance steroids | 18 patients included |
| 54 | Piga M, et al. 2013: Rituximab treatment for “rhupus syndrome”: clinical and power-Doppler ultrasonographic monitoring of response | 6 patients included |
| 55 | Pinto CM et al., 203: Bone mineral density in systemic lupus erythematosus women one year after rituximab therapy | No prognostic factor analysis |
| 56 | Pinto LF et al., 2011: Rituximab induces a rapid and sustained remission in Colombian patients with severe and refractory systemic lupus erythematosus | No prognostic factor analysis |
| 57 | Podolskaya A et al., 2008: B cell depletion therapy for 19 patients with refractory systemic lupus erythematosus | No prognostic factor analysis and 19 patients included |
| 58 | Pusongchai T et al., 2010: Pediatric systemic lupus erythematosus in Thammasat University Hospital | 19 patients included |
| 59 | Ramos-Casals M et al., 2009: Rituximab in systemic lupus erythematosus A systematic review of off-label use in 188 cases | Systematic review |
| 60 | Ramos-Casals M et al., 2010: Off-label use of rituximab in 196 patients with severe, refractory systemic autoimmune diseases | No prognostic factor analysis |
| 61 | Reddy V et al., 2015: Internalization of rituximab and the efficiency of B cell depletion in rheumatoid arthritis and systemic lupus erythematosus | In vitro experiment |
| 62 | Reynolds JA et al. 2009: Effects of rituximab on resistant SLE disease including lung involvement | 11 patients included |
| 63 | Roberts DM et al., 2015: Rituximab-associated hypogammaglobulinemia: Incidence, predictors and outcomes in patients with multi-system autoimmune disease | No prognostic factors analysis explored for SLE patients |
| 64 | Roccatello D et al., 2011: Intensive short-term treatment with rituximab, cyclophosphamide and methylprednisolone pulses induces remission in severe cases of SLE with nephritis and avoids further immunosuppressive maintenance therapy. | 8 patients included |
| 65 | Scheinberg M et al., 2006: Rituximab in refractory autoimmune diseases: Brazilian experience with 29 patients (2002-2004) | 5 patients included |
| 66 | Sfikakis PP et al., 2005: Remission of proliferative lupus nephritis following B cell depletion therapy is preceded by down-regulation of the T cell costimulatory molecule CD40 ligand. An open-label trial | 10 patients included |
| 67 | Sifikakis PP et al., 2009: Clonal expansion of B-cells in human systemic lupus erythematosus: evidence from studies before and after therapeutic B-cell depletion. | 7 patients included |
| 68 | Tanaka Y et al., 2015: Efficacy and safety of rituximab in Japanese patients with systemic lupus erythematosus and lupus nephritis who are refractory to conventional therapy | No prognostic factor analysis |
| 69 | Terrier B et al., 2010: Safety and efficacy of rituximab in systemic lupus erythematosus. Results from 136 patients from the French AutoImmunity and Rituximab Registry | No baseline prognostic factors |
| 70 | Tokunaga M et al., 2005: Down-regulation of CD40 and CD80 on B cells in patients with life-threatening systemic lupus erythematosus after successful treatment with rituximab | 5 patients included |
| 71 | Tokunaga M et al., 2007: Efficacy of rituximab (anti-CD20) for refractory systemic lupus erythematosus involving the central nervous system | 10 patients included |
| 72 | Tony HP et al., 2011: Safety and clinical outcomes of rituximab therapy in patients with different autoimmune diseases: experience from a national registry (GRAID). | No prognostic factor analysis |
| 73 | Turner-Stokes T et al., 2011: The efficacy of repeated treatment with B-cell depletion therapy in systemic lupus erythematosus: an evaluation | 18 patients included |
| 74 | Vigna-Perez M et al., 2006: Clinical and immunological effects of rituximab in patients with lupus nephritis refractory to conventional therapy: a pilot study. | 22 patients included |
| 75 | Weidenbusch M et al., 2009: Beyond the LUNAR trial. Efficacy of rituximab in refractory lupus nephritis | Systematic review |
| 76 | Willems M et al., 2006: Rituximab therapy for childhood-onset systemic lupus erythematosus. | 11 patients included |
| 77 | Witt M et al., 2013: Clinical outcomes and safety of rituximab treatment for patients with systemic lupus erythematosus (SLE) – results from a nationwide cohort in Germany (GRAID) | No prognostic factor analysis |

**Supplementary file B**

**Table B.1 Methodological Quality of Subgroup Analysis**

| **Quality appraisal for**  **sub-group studies** | **1. Was the**  **analysis**  **a priori?** | **2. Was selection**  **of factors for**  **analysis theory/**  **evidence driven** | **3. Were sub-groups**  **measured prior to**  **randomization?** | **4. Adequate**  **quality of**  **measurement**  **of baseline**  **factors?** | **5. Contains an explicit**  **test of the interaction**  **between sub-group**  **and treatment**  **(e.g., regression)?** | **Strength of**  **evidence** |
| --- | --- | --- | --- | --- | --- | --- |
| Merrill et al. 2010 | Yes | Unclear | Yes | Unclear | No | Exploratory |
| Tew et al 2010 | No | No | Yes | Yes | No | Insufficient |

**Confirmatory evidence:** The study fulfils all of the quality assessment criteria for moderator studies (a priori analysis, factors evidence driven, moderators measured prior to randomization, adequate measurement of baseline factors and explicit test of the interaction between moderator and treatment). **Exploratory evidence:** Fulfilling the last three quality assessment criteria. **Insufficient evidence:** The study did not carry out an explicit test of interaction or measurement of the sub-groups was reported to take place post randomization.

**Table B.2 Risk of bias of the included studies (n=14)**

| Study | **Study participation:**  The study sample  adequately represents the  population of interest | **Study attrition:**  The study data from participants not lost to follow-up accurately  represent the sample | **Prognostic factor measurement:**  The prognostic/risk  factor is valid and  measured in a similar way for all participants | **Outcome measurement:**  The outcome is valid and measured in a similar way for all participants | **Study confounding:**  Important potential  Confounding factors are appropriately accounted | **Statistical analysis and reporting:**  The statistical analysis is appropriate, and all primary outcomes are reported | **Risk of**  **bias**  **assessment** |
| --- | --- | --- | --- | --- | --- | --- | --- |
| Carter 2013 | High | High | Moderate | Low | High | High | - |
| Catapano 2010 | Moderate | Moderate | Moderate | Low | High | High | - |
| Dias 2015 | High | Moderate | Low | Low | Moderate | Moderate | +/- |
| Fernandez-Nebro 2012 | Moderate | Moderate | Moderate | Low | Moderate | Moderate | +/- |
| Jónsdóttir 2010 | High | Moderate | Moderate | High | High | Moderate | - |
| Lazarus 2012 | High | High | Moderate | Low | High | Moderate | - |
| Lindholm  2008 | Moderate | Moderate | Moderate | Low | High | High | - |
| Marquez 2013 | High | Moderate | Moderate | Moderate | Moderate | Moderate | +/- |
| Ng 2007 | High | Moderate | Moderate | Low | Moderate | Moderate | +/- |
| Robledo 2012 | High | Moderate | Moderate | High | High | High | - |
| Robledo 2012 | High | Moderate | Moderate | High | Moderate | Moderate | - |
| Vital 2011 | High | High | Moderate | Low | Moderate | Moderate | +/- |
| Vital 2015 | Moderate | Moderate | Moderate | Low | High | Moderate | +/- |
|  |  |  |  |  |  |  |  |

Low = Low risk of bias; Moderate = Moderate risk of bias; High = High risk of bias. Overall methodological quality: + = high; +/- = moderate; - = low

**Supplementary file C**

**GRADE profiles for Intervention studies.**

**PICO 1.** In SLE patients with sociodemographic factors (Different races) (P), what is the effect of Rituximab (I) on outcome (O) compared with control (C)?

**Control: Placebo**

| **Quality assessment** | | | | | | | **No of patients** | | **Effect** | | **Quality** |
| --- | --- | --- | --- | --- | --- | --- | --- | --- | --- | --- | --- |
|  |  |  |  |  |  |  |  |  |  |  |  |
| **No of studies** | **Design** | **Risk of bias** | **Inconsistency** | **Indirectness** | **Imprecision** | **Other considerations** | **Rituximab** | **Placebo** | **Relative (95% CI)** | **Absolute** |  |
| **Major clinical response (follow-up 52 weeks; assessed with: criteria) in African American/Hispanic population** | | | | | | | | | | | |
| 1 (Merrill 2010) | randomised trials | serious^1,2,3^ | no serious inconsistency | serious^4^ | very serious^5,6^ | none | 9/65  (13.8%) | 3/32  (9.4%) | RR 1.47 (0.42 to 5.08) | 44 more per 1000 (from 54 fewer to 382 more) | ⊕OOO VERY LOW |
| **Major clinical response (follow-up 52 weeks; assessed with: criteria) in White, Asian/Pacific Islander and Others** | | | | | | | | | | | |
| 1 (Merrill 2010) | randomised trials | serious^1,2,3^ | no serious inconsistency | serious^4^ | very serious^5,6^ | none | 12/104  (11.5%) | 11/56  (19.6%) | RR 1.10 (0.5 to 2.44) | 20 more per 1000 (from 98 fewer to 283 more) | ⊕OOO VERY LOW |
| **Partial clinical response (follow-up 52 weeks; assessed with: criteria) in African American/Hispanic population** | | | | | | | | | | | |
| 1 (Merrill 2010) | randomised trials | serious^1,2,3^ | no serious inconsistency | serious^4^ | serious^5^ | none | 13/65  (20%) | 2/32  (6.3%) | RR 3.2 (0.76 to 13.3) | 138 more per 1000 (from 15 fewer to 769 more) | ⊕OOO VERY LOW |
| **Partial clinical response (follow-up 52 weeks; assessed with: criteria) in White, Asian/Pacific Islander and Others** | | | | | | | | | | | |
| 1 (Merrill 2010) | randomised trials | serious^1,2,3^ | no serious inconsistency | serious^4^ | serious^5,6^ | none | 16/104  (15.4%) | 9/56  (16.1%) | RR 0.95 (0.45 to 2.02) | 8 fewer per 1000 (from 88 fewer to 164 more) | ⊕OOO VERY LOW |
| **Overall response (follow-up 52 weeks; assessed with: Major + Partial response) in African American/Hispanic population** | | | | | | | | | | | |
| 1 (Merrill 2010) | randomised trials | serious^1,2,3^ | no serious inconsistency | serious^4^ | serious^5^ | none | 22/65  (33.8%) | 5/32  (15.6%) | RR 2.16 (0.9 to 5.19) | 181 more per 1000 (from 16 fewer to 655 more) | ⊕OOO VERY LOW |
| **Overall response (follow-up 52 weeks; assessed with: Major + Partial response) in White, Asian/Pacific Islander and Others** | | | | | | | | | | | |
| 1 (Merrill 2010) | randomised trials | very serious^1,2,3^ | no serious inconsistency | serious^4^ | serious^5^ | none | 28/104  (26.9%) | 20/56  (35.7%) | RR 0.75 (0.46 to 1.2) | 89 fewer per 1000 (from 193 fewer to 71 more) | ⊕OOO VERY LOW |
| **No clinical response (follow-up 52 weeks; assessed with: criteria) in African American/Hispanic population** | | | | | | | | | | | |
| 1 (Merrill 2010) | randomised trials | serious^1,2,3^ | no serious inconsistency | serious^4^ | serious^5^ | none | 43/65  (66.2%) | 27/32  (84.4%) | **RR 0.78 (0.62 to 0.98)** | 186 fewer per 1000 (from 17 fewer to 321 fewer) | ⊕OOO VERY LOW |
| **No clinical response (follow-up 52 weeks; assessed with: criteria) in White, Asian/Pacific Islander and Others** | | | | | | | | | | | |
| 1 (Merrill 2010) | randomised trials | serious^1,2,3^ | no serious inconsistency | serious^4^ | serious^5^ | none | 76/104 (73.1%) | 36/56 (64.3%) | RR 1.3 (0.9 to 1.42) | 84 more per 1000 (from fewer 64 to more 270) | ⊕OOO VERY LOW |

^1^ allocation concealment unclear; ^2^ sequence generation for randomization is unclear; ^3^ post hoc subgroup analysis; ^4^ Related to the population, because race groups are very heterogeneous; ^5^ does not meet "optimal information size" (i.e. insufficient sample size according to post-hoc power calculation); ^6^ confidence interval does neither exclude Relative Risk (RR) reduction nor RR increase by 50%.

**Supplementary file D**

**Table D.1 Quality of evidence according to GRADE (outcome: overall clinical response)**

| **D.GRADE factors** | | | | | | | | | | | | | | | | | | |
| --- | --- | --- | --- | --- | --- | --- | --- | --- | --- | --- | --- | --- | --- | --- | --- | --- | --- | --- |
| **Factor identified** | **N studies** | **N cohorts** | **Univariate analysis** | | | **Multivariate analysis** | | | | **Phase** | **Study limitations** | **Inconsistency** | **Indirectness** | **Imprecision** | **Publication bias** | **Moderate-large effect size** | **Exposure-response gradient** | **Overall quality** |
|  |  |  | + | 0 | - | + | 0 | | - |  |  |  |  |  |  |  |  |  |
| - 174 IL-6 (rs1800795) SNP CC genotype [34] | 1 | 1 | 0 | 1 | 0 |  |  | |  | 1 | X | ✓ | ✓ | X | X | X | X | 🞣 |
| - 174 IL-6 (rs1800795) SNP GC genotype [34] | 1 | 1 | 0 | 1 | 0 |  |  | |  | 1 | X | ✓ | ✓ | X | X | X | X | 🞣 |
| - 174 IL-6 (rs1800795) SNP GG genotype [34] | 1 | 1 | 0 | 1 | 0 |  |  | |  | 1 | X | ✓ | ✓ | X | X | X | X | 🞣 |
| FCGR3A-158 SNP FF genotype [35] | 1 | 1 | 0 | 1 | 0 |  |  | |  | 1 | X | ✓ | ✓ | X | X | X | X | 🞣 |
| FCGR3A-158 SNP FV genotype [35] | 1 | 1 | 0 | 1 | 0 |  |  | |  | 1 | X | ✓ | ✓ | X | X | X | X | 🞣 |
| FCGR3A-158 SNP VV genotype [35] | 1 | 1 | 0 | 1 | 0 |  |  | |  | 1 | X | ✓ | ✓ | X | X | X | X | 🞣 |
| IL2/IL21 SNP (rs6822844) GG genotype [36] | 1 | 1 | 0 | 1 | 0 | 1 | 0 | | 0 | 2 | X | ✓ | ✓ | X | X | ✓ | X | 🞣🞣 |
| IL2/IL21 SNP (rs6822844) G allele [36] | 1 | 1 | 1 | 0 | 0 |  |  | |  | 2 | X | ✓ | ✓ | X | X | X | X | 🞣 |
| Baseline SLEDAI [31] | 1 | 1 | 1 | 0 | 0 | 1 | | 0 | 0 | 1 | X | ✓ | ✓ | ✓ | X | X | X | 🞣 |
| Previous discoid rash [31] | 1 | 1 | 1 | 0 | 0 | 0 | | 1 | 0 | 1 | X | ✓ | ✓ | ✓ | X | X | X | 🞣 |
| Previous severe hematologic disorder [31] | 1 | 1 | 0 | 0 | 1 | 0 | | 0 | 1 | 1 | X | ✓ | ✓ | ✓ | X | ✓ | X | 🞣 |
| Previous treatment with immunoglobulins [31] | 1 | 1 | 1 | 0 | 0 | 0 | | 1 | 0 | 1 | X | ✓ | ✓ | ✓ | X | X | X | 🞣 |
| Previous treatment with prednisolone ≥ 100 mg/day [31] | 1 | 1 | 1 | 0 | 0 | 1 | | 0 | 0 | 1 | X | ✓ | ✓ | ✓ | X | ✓ | X | 🞣 |
| Longer duration of BCD [30] | 1 | 1 | 1 | 0 | 0 |  | |  |  | 1 | X | ✓ | ✓ | X | X | X | X | 🞣 |
| Complete B depletion [13] | 1 | 1 | 1 | 0 | 0 |  | |  |  | 1 | X | ✓ | ✓ | X | X | X | X | 🞣 |

Phase, phase of investigation: phase 1 explanatory study, identifying associations; phase 2 explanatory study, testing independent associations; phase 3 explanatory study, understanding prognostic pathways

For uni- and multivariate analyses: +, number of significant effects with a positive value; 0, number of non-significant effects; -, number of significant effects with a negative value.

For GRADE factors: ✓, no serious limitations; X, serious limitations (or not present for moderate/large effect size, dose effect); unclear, unable to rate item based on available information. For overall quality of evidence: +, very low; ++, low; +++, moderate; ++++, high

**Abbreviations:** BCD: B cell depletion; GRADE: Grading of Recommendations Assessment, Development and Evaluation; SELENA-SLEDAI: Safety of Estrogens in Lupus Erythematosus National Assessment-SLE disease activity index; SNP: single nucleotide polymorphism.

**Table D.2 Quality of evidence according to GRADE (outcome: overall clinical relapse or flare)**

| **GRADE factors** | | | | | | | | | | | | | | | | | |
| --- | --- | --- | --- | --- | --- | --- | --- | --- | --- | --- | --- | --- | --- | --- | --- | --- | --- |
| **Factor identified** | **N studies** | **N cohorts** | **Univariate analysis** | | | **Multivariate analysis** | | | **Phase** | **Study limitations** | **Inconsistency** | **Indirectness** | **Imprecision** | **Publication bias** | **Moderate-large effect size** | **Exposure-response gradient** | **Overall quality** |
|  |  |  | + | 0 | - | + | 0 | - |  |  |  |  |  |  |  |  |  |
| Baseline anti-DNA [27;29] | 2 | 2 | 0 | 2 | 0 |  |  |  | 1 | X | ✓ | ✓ | X | X | X | X | 🞣 |
| Baseline anti-ENA [13;29;33] | 3 | 3 | 2 | 1 | 0 | 1 | 0 | 0 | 1 | X | X | ✓ | X | ✓ | X | X | 🞣 |
| Baseline BAFF [26] | 1 | 1 | 0 | 1 | 0 |  |  |  | 1 | X | ✓ | ✓ | X | X | X | X | 🞣 |
| BAFF levels after BCD [26] | 1 | 1 | 1 | 0 | 0 |  |  |  | 1 | X | ✓ | ✓ | X | X | X | X | 🞣 |
| B cell repopulation [13] | 1 | 1 | 1 | 0 | 0 |  |  |  | 1 | X | ✓ | ✓ | X | X | X | X | 🞣 |
| B cell repopulation with higher anti-DNA [27] | 1 | 1 | 1 | 0 | 0 |  |  |  | 1 | X | ✓ | ✓ | X | X | X | X | 🞣 |

Phase, phase of investigation: phase 1 explanatory study, identifying associations; phase 2 explanatory study, testing independent associations; phase 3 explanatory study, understanding prognostic pathways

For uni- and multivariate analyses: +, number of significant effects with a positive value; 0, number of non-significant effects; -, number of significant effects with a negative value.

For GRADE factors: ✓, no serious limitations; X, serious limitations (or not present for moderate/large effect size, dose effect); unclear, unable to rate item based on available information. For overall quality of evidence: +, very low; ++, low; +++, moderate; ++++, high

**Abbreviations:** BAFF: B-cell–activating factor; anti-ENA: anti-extractable nuclear antigen; BCD: B cell depletion; GRADE: Grading of Recommendations Assessment, Development and Evaluation.

**Table D.3 Quality of evidence according to GRADE (outcome: cutaneous response)**

| **GRADE factors** | | | | | | | | | | | | | | | | | |
| --- | --- | --- | --- | --- | --- | --- | --- | --- | --- | --- | --- | --- | --- | --- | --- | --- | --- |
| **Factor identified** | **N studies** | **N cohorts** | **Univariate analysis** | | | **Multivariate analysis** | | | **Phase** | **Study limitations** | **Inconsistency** | **Indirectness** | **Imprecision** | **Publication bias** | **Moderate-large effect size** | **Exposure-response gradient** | **Overall quality** |
|  |  |  | + | 0 | - | + | 0 | - |  |  |  |  |  |  |  |  |  |
| Subtype skin disease [37] | 1 | 1 | 0 | 1 | 0 |  |  |  | 1 | X | ✓ | ✓ | X | X | X | X | 🞣 |
| Baseline anti-Ro/SSA antibodies [37] | 1 | 1 | 1 | 0 | 0 |  |  |  | 1 | X | ✓ | ✓ | X | X | X | X | 🞣 |
| Baseline anti-La antibodies [37] | 1 | 1 | 0 | 1 | 0 |  |  |  | 1 | X | ✓ | ✓ | X | X | X | X | 🞣 |
| Baseline anti-dsDNA antibodies [37] | 1 | 1 | 0 | 1 | 0 |  |  |  | 1 | X | ✓ | ✓ | X | X | X | X | 🞣 |
| Baseline anti-Sm antibodies [37] | 1 | 1 | 0 | 1 | 0 |  |  |  | 1 | X | ✓ | ✓ | X | X | X | X | 🞣 |
| Baseline anti-RNP antibodies [37] | 1 | 1 | 0 | 1 | 0 |  |  |  | 1 | X | ✓ | ✓ | X | X | X | X | 🞣 |
| Baseline complement [37] | 1 | 1 | 0 | 1 | 0 |  |  |  | 1 | X | ✓ | ✓ | X | X | X | X | 🞣 |
| Complete B depletion [37] | 1 | 1 | 0 | 1 | 0 |  |  |  | 1 | X | ✓ | ✓ | X | X | X | X | 🞣 |

Phase, phase of investigation: phase 1 explanatory study, identifying associations; phase 2 explanatory study, testing independent associations; phase 3 explanatory study, understanding prognostic pathways

For uni- and multivariate analyses: +, number of significant effects with a positive value; 0, number of non-significant effects; -, number of significant effects with a negative value.

For GRADE factors: ✓, no serious limitations; X, serious limitations (or not present for moderate/large effect size, dose effect); unclear, unable to rate item based on available information. For overall quality of evidence: +, very low; ++, low; +++, moderate; ++++, high

**Abbreviations:** anti-RNP: anti-ribonucleoprotein; GRADE: Grading of Recommendations Assessment, Development and Evaluation; HCQ: hydroxychloroquine

**Table D.4 Quality of evidence according to GRADE (outcome: cutaneous relapse or flare).**

| **GRADE factors** | | | | | | | | | | | | | | | | | | |
| --- | --- | --- | --- | --- | --- | --- | --- | --- | --- | --- | --- | --- | --- | --- | --- | --- | --- | --- |
| **Factor identified** | **N studies** | **N cohorts** | **Univariate analysis** | | | **Multivariate analysis** | | | | **Phase** | **Study limitations** | **Inconsistency** | **Indirectness** | **Imprecision** | **Publication bias** | **Moderate-large effect size** | **Exposure-response gradient** | **Overall quality** |
|  |  |  | + | 0 | - | + | 0 | | - |  |  |  |  |  |  |  |  |  |
| Subtype skin disease [37] | 1 | 1 | 0 | 1 | 0 |  |  | |  | 1 | X | ✓ | ✓ | X | X | X | X | 🞣 |
| Baseline anti-Ro/SSA antibodies [37] | 1 | 1 | 0 | 1 | 0 |  |  | |  | 1 | X | ✓ | ✓ | X | X | X | X | 🞣 |
| Baseline anti-La antibodies [37] | 1 | 1 | 0 | 1 | 0 |  |  | |  | 1 | X | ✓ | ✓ | X | X | X | X | 🞣 |
| Baseline anti-dsDNA antibodies [37] | 1 | 1 | 0 | 1 | 0 |  |  | |  | 1 | X | ✓ | ✓ | X | X | X | X | 🞣 |
| Baseline anti-Sm antibodies [37] | 1 | 1 | 0 | 1 | 0 |  |  | |  | 1 | X | ✓ | ✓ | X | X | X | X | 🞣 |
| Baseline anti-RNP antibodies [37] | 1 | 1 | 0 | 1 | 0 |  |  | |  | 1 | X | ✓ | ✓ | X | X | X | X | 🞣 |
| Baseline complement [37] | 1 | 1 | 0 | 1 | 0 |  |  | |  | 1 | X | ✓ | ✓ | X | X | X | X | 🞣 |
| Complete B depletion [37] | 1 | 1 | 0 | 1 | 0 |  |  |  | | 1 | X | ✓ | ✓ | X | X | X | X | 🞣 |

Phase, phase of investigation: phase 1 explanatory study, identifying associations; phase 2 explanatory study, testing independent associations; phase 3 explanatory study, understanding prognostic pathways

For uni- and multivariate analyses: +, number of significant effects with a positive value; 0, number of non-significant effects; -, number of significant effects with a negative value.

For GRADE factors: ✓, no serious limitations; X, serious limitations (or not present for moderate/large effect size, dose effect); unclear, unable to rate item based on available information. For overall quality of evidence: +, very low; ++, low; +++, moderate; ++++, high

**Abbreviations**: GRADE: Grading of Recommendations Assessment, Development and Evaluation

**Table D.5 Quality of evidence according to GRADE (outcome: renal response)**

| **GRADE factors** | | | | | | | | | | | | | | | | | |
| --- | --- | --- | --- | --- | --- | --- | --- | --- | --- | --- | --- | --- | --- | --- | --- | --- | --- |
| **Factor identified** | **N studies** | **N cohorts** | **Univariate analysis** | | | **Multivariate analysis** | | | **Phase** | **Study limitation** | **Inconsistency** | **Indirectness** | **Imprecision** | **Publication bias** | **Moderate-large effect size** | **Exposure-response gradient** | **Overall quality** |
|  |  |  | + | 0 | - | + | 0 | - |  |  |  |  |  |  |  |  |  |
| Duration of LN [28] | 1 | 1 | 0 | 1 | 0 |  |  |  | 1 | X | ✓ | ✓ | X | X | X | X | 🞣 |
| LN class. [32] | 1 | 2 | 0 | 1 | 0 |  |  |  | 1 | X | ✓ | ✓ | X | X | X | X | 🞣 |
| Baseline Serum creatinine [28] | 1 | 1 | 0 | 0 | 1 |  |  |  | 1 | X | ✓ | ✓ | X | X | X | X | 🞣 |
| Baseline eGFR < 30 ml/min [28] | 1 | 1 | 0 | 1 | 0 |  |  |  | 1 | X | ✓ | ✓ | X | X | X | X | 🞣 |
| Baseline proteinuria [28] | 1 | 1 | 0 | 1 | 0 |  |  |  | 1 | X | ✓ | ✓ | X | X | X | X | 🞣 |
| Baseline anti-dsDNA antibodies [28] | 1 | 1 | 0 | 1 | 0 |  |  |  | 1 | X | ✓ | ✓ | X | X | X | X | 🞣 |
| Baseline C3 levels [28] | 1 | 1 | 0 | 1 | 0 |  |  |  | 1 | X | ✓ | ✓ | X | X | X | X | 🞣 |
| Baseline detectable circulating CD19+B cells [28] | 1 | 1 | 0 | 1 | 0 |  |  |  | 1 | X | X | ✓ | X | X | X | X | 🞣 |

Phase, phase of investigation: phase 1 explanatory study, identifying associations; phase 2 explanatory study, testing independent associations; phase 3 explanatory study, understanding prognostic pathways

For uni- and multivariate analyses: +, number of significant effects with a positive value; 0, number of non-significant effects; -, number of significant effects with a negative value.

For GRADE factors: ✓, no serious limitations; X, serious limitations (or not present for moderate/large effect size, dose effect); unclear, unable to rate item based on available information. For overall quality of evidence: +, very low; ++, low; +++, moderate; ++++, high

**Abbreviations:** eGFR; estimated glomerular filtration; GRADE: Grading of Recommendations Assessment, Development and Evaluation; LN: lupus nephritis.

**Table D.6 Quality of evidence according to GRADE (outcome: side effects)**

| **GRADE factors** | | | | | | | | | | | | | | | | | |
| --- | --- | --- | --- | --- | --- | --- | --- | --- | --- | --- | --- | --- | --- | --- | --- | --- | --- |
| **Possible predictors** | **N studies** | **N cohorts** | **Univariate analysis** | | | **Multivariate analysis** | | | **Phase** | **Study limitations** | **Inconsistency** | **Indirectness** | **Imprecision** | **Publication bias** | **Moderate-large effect size** | **Exposure-response gradient** | **Overall quality** |
|  |  |  | + | 0 | - | + | 0 | - |  |  |  |  |  |  |  |  |  |
| Comorbidity [31] | 1 | 1 | 1 | 0 | 0 | 1 | 0 | 0 | 1 | X | ✓ | ✓ | ✓ | X | X | X | 🞣 |
| No. of severely involved organ systems [31] | 1 | 1 | 1 | 0 | 0 | 1 | 0 | 0 | 1 | X | ✓ | ✓ | ✓ | X | ✓ | X | 🞣🞣 |
| Baseline leukocyte count, x10^9^/L [31] | 1 | 1 | 1 | 0 | 0 | 1 | 0 | 0 | 1 | X | ✓ | ✓ | ✓ | X | X | X | 🞣 |
| Complete BCD [13] | 1 | 1 | 0 | 1 | 0 |  |  |  | 1 | X | ✓ | ✓ | X | X | X | X | 🞣 |
| Previous treatment with steroid bolus [31] | 1 | 1 | 1 | 0 | 0 | 1 | 0 | 0 | 1 | X | ✓ | ✓ | ✓ | X | ✓ | X | 🞣🞣 |

Phase, phase of investigation: phase 1 explanatory study, identifying associations; phase 2 explanatory study, testing independent associations; phase 3 explanatory study, understanding prognostic pathways

For uni- and multivariate analyses: +, number of significant effects with a positive value; 0, number of non-significant effects; -, number of significant effects with a negative value.

For GRADE factors: ✓, no serious limitations; X, serious limitations (or not present for moderate/large effect size, dose effect); unclear, unable to rate item based on available information. For overall quality of evidence: +, very low; ++, low; +++, moderate; ++++, high

**Abbreviations:** BCD: B cell depletion.

**Table D.7 Quality of evidence according to GRADE (outcome: changes in biomarkers)**

| **GRADE factors** | | | | | | | | | | | | | | | | | |
| --- | --- | --- | --- | --- | --- | --- | --- | --- | --- | --- | --- | --- | --- | --- | --- | --- | --- |
| **Possible predictors** | **N studies** | **N cohorts** | **Univariate analysis** | | | **Multivariate analysis** | | | **Phase** | **Study limitations** | **Inconsistency** | **Indirectness** | **Imprecision** | **Publication bias** | **Moderate-large effect size** | **Exposure-response gradient** | **Overall quality** |
|  |  |  | + | 0 | - | + | 0 | - |  |  |  |  |  |  |  |  |  |
| Baseline high-DNA [9;13] | 2 | 1 | 1 | 1 | 0 |  |  |  | 1 | X | X | ✓ | X | X | X | X | 🞣 |
| Baseline anti-dsDNA^+^RNP^-^ | 1 | 1 | 1 | 0 | 0 |  |  |  | 1 | X | ✓ | ✓ | X | X | X | X | 🞣 |
| Baseline BAFF [25] | 1 | 1 | 0 | 1 | 0 |  |  |  | 1 | X | ✓ | ✓ | X | X | X | X | 🞣 |
| Baseline anti-ENA antibodies [13] | 1 | 1 | 0 | 1 | 0 |  |  |  | 1 | X | ✓ | ✓ | X | X | X | X | 🞣 |
| Baseline low C3 or C4 [13] | 1 | 1 | 0 | 1 | 0 |  |  |  | 1 | X | ✓ | ✓ | X | X | X | X | 🞣 |
| Baseline memory cells [13] | 1 | 1 | 1 | 0 | 0 |  |  |  | 1 | X | ✓ | ✓ | X | X | X | X | 🞣 |
| Baseline plasmablast cells [13] | 1 | 1 | 0 | 0 | 0 |  |  |  | 1 | X | ✓ | ✓ | X | X | X | X | 🞣 |
| Baseline high BAFF [25] | 1 | 1 | 0 | 0 | 1 |  |  |  | 1 | X | ✓ | ✓ | X | X | X | X | 🞣 |
| Baseline BAFF levels [26] | 2 | 2 | 1 | 1 | 0 |  |  |  | 1 | X | ✓ | ✓ | X | X | X | X | 🞣 |
| Baseline IFN signature [25] | 1 | 1 | 0 | 1 | 0 |  |  |  | 1 | X | ✓ | ✓ | X | X | X | X | 🞣 |

Phase, phase of investigation: phase 1 explanatory study, identifying associations; phase 2 explanatory study, testing independent associations; phase 3 explanatory study, understanding prognostic pathways

For uni- and multivariate analyses: +, number of significant effects with a positive value; 0, number of non-significant effects; -, number of significant effects with a negative value.

**Abbreviatio**ns: BAFF: lymphocyte stimulator; GRADE: Grading of Recommendations Assessment, Development and Evaluation; IFN: interferon.
